# Integrative Epigenomics: Bioinformatics Strategies for Multi-Omics Data Analysis in Health and Disease

**DOI:** 10.3390/epigenomes10030053

**Published:** 2026-08-07

**Authors:** Shikhi Baruri, Lalit Batra, Sohome Adhikari, Ayman El-Baz

**Affiliations:** 1 Center for Predictive Medicine for Biodefense and Emerging Infectious Diseases (CPM), University of Louisville, Louisville, KY 40222, USA; 2 Bioengineering Department, J.B. Speed School of Engineering, University of Louisville, Louisville, KY 40292, USA; aselba01@louisville.edu; 3 Department of Computer Science and Engineering, University of Louisville, Louisville, KY 40292, USA; sohome.adhikari@louisville.edu

**Keywords:** epigenomics, histone modification, DNA methylation, ChIP-seq, ATAC-seq, bioinformatics tools, epigenetic regulation, multi-omics integration, single-cell epigenomics, machine learning, clinical epigenetic biomarkers

## Abstract

**Background:** Epigenomics has emerged as an essential field in modern molecular biology, providing a critical layer of gene regulation. DNA methylation, histone modifications and alterations to the chromatin accessibility of DNA have been widely associated with complex diseases including cancer. The most recent developments in high-throughput sequencing technology have made it possible to profile epigenetic landscapes genomically on a large scale. However, bulk averaging can obscure cellular heterogeneity essential for understanding complex disease states. The purpose of the review is to survey accessible tools and algorithms to conduct an Epigenomic study in the field of biomedical research, from bulk tissue analysis to the high-resolution frontier of single-cell epigenomics. **Methods:** We performed a comparative analysis of common methods used to analyze DNA methylation, chromatin immunoprecipitation, sequencing analysis and chromatin accessibility profiling. We described the standardized bioinformatics tools and pipelines required to transform raw sequencing data into mechanistic biological understanding, highlighting the role of quality control, peak calling, and differential analysis. Furthermore, we explore the integration of epigenomics with other “omics” layers through advanced computational frameworks, including machine learning and network-based modeling. **Results:** These advanced multi-omics techniques demonstrate promising clinical utility by enabling biomarker discovery, disease subtyping, and identification of novel therapeutic targets. **Conclusions:** Despite challenges with data complexity, the fusion of Artificial Intelligence (AI) and single-cell technologies will accelerate the transition toward precision medicine.

## 1. Introduction

Epigenomics is the study of the whole genome, focusing on DNA methylation, histone modifications, chromatin structure, and noncoding RNAs. These mechanisms modulate gene expression without altering the underlying DNA sequence [1,2]. These epigenetic mechanisms are hereditary and reversible, and they are essential for regulating cellular identity, development, and tissue-specific functions by controlling the switches that turn genes on or off in different cellular contexts [3]. Consequently, abnormalities in epigenetic marks can cause complex diseases, such as cancer, cardiovascular, metabolic, and immune-related diseases [1,4,5,6,7].

Recently, next-generation sequencing (NGS) has accelerated epigenomic discovery by enabling genome-scale profiling of DNA methylation, histone modifications, and chromatin accessibility [8]. These advancements have facilitated the identification of epigenetic biomarkers in precision medicine. In fields such as oncology and cardiometabolic research, drugs that target epigenetic mechanisms are currently being explored to reverse disease-associated alterations. Furthermore, the emergence of single-cell epigenomics has provided a powerful approach to decipher cellular heterogeneity and plasticity, offering critical insights into stem cell biology and tumor heterogeneity [3].

However, the rapid expansion of epigenomic platforms has created a need to evaluate the performance of computational tools across varied data types and analytical objectives. Specifically, challenges related to data quality, normalization, peak detection, functional annotation, batch effects, single-cell data sparsity, and cross-platform integration still impede reproducibility and translational applications. These obstacles are particularly pronounced in multi-omics research, where epigenomic data must be integrated with transcriptomic, genomic, proteomic, or clinical datasets.

There have been several recent reviews on particular aspects of epigenomic bioinformatics. For example, Benčik et al. [9] reviewed experimental techniques for DNA methylation, histone modifications, and non-coding RNAs, with a primary focus on wet-lab techniques and limited attention to computational pipelines and data integration. Cheng et al. [10] provided a comprehensive benchmarking assessment of bioinformatics strategies for Tn5-based chromatin accessibility assays, but did not address DNA methylation analysis, ChIP-seq analysis, single-cell frameworks, multi-omics integration, or clinical translation. Lin and Liu [11] reviewed advances in epigenomic sequencing technologies and how these technologies could be used for cancer diagnostics, without addressing the computational and analytical decisions required to translate raw data into biological inference. The aim of this review is to fill this gap and provide a critical examination of current epigenetic analysis tools, encompassing experimental platforms, computational pipelines, and commercially available technologies. We further explore machine learning strategies, including matrix factorization, network-based fusion, and deep learning, to enhance pattern recognition, biomarker discovery, and disease prediction [12]. By reducing dimensionality and identifying cross-modal relationships, these techniques allow researchers to extract meaningful biological insights from high-throughput assays. We also explore the role of epigenomics in cancer and other complex diseases, with particular emphasis on how epigenomic profiling is being implemented into early detection, diagnosis and therapeutic decision-making. Lastly, this review critically evaluates the applications of the tools, their drawbacks, and their suitability for different epigenomic and multi-omics study designs. We also highlight new trends and future directions in the domain along with a clear view of how epigenomic research is defining the next generation of precision medicine.

## 2. Methodology of Literature Search

This study was conducted as a narrative review to provide a critical and representative overview of current bioinformatics approaches to epigenomic and multi-omics research. In this study, we conducted a comprehensive literature review spanning the last decade using electronic databases, including PubMed and Google Scholar. The search covered articles published from January 2015 to March 2026. Articles were filtered out if they were not published in English, were not directly related to epigenomic analysis or computational integration, or lacked sufficient methodological detail for meaningful discussion.

The following keywords and their combinations were used: “Epigenomics”, “Histone modification”, “DNA methylation”, “ChIP-seq”, “ATAC-seq”, “Bioinformatics tools”, “Epigenetic regulation”, “Multi-omics integration”, “Single-cell epigenomics”, “Machine learning”, “epigenetic biomarkers”, “scATAC-seq”, “Principal component analysis (PCA) and canonical correlation analysis (CCA)”, “Matrix Factorization”, “Computational integration strategies”, “network-based methods”, “autoencoder”, “variational autoencoder (VAE)”.

**Figure 1:**
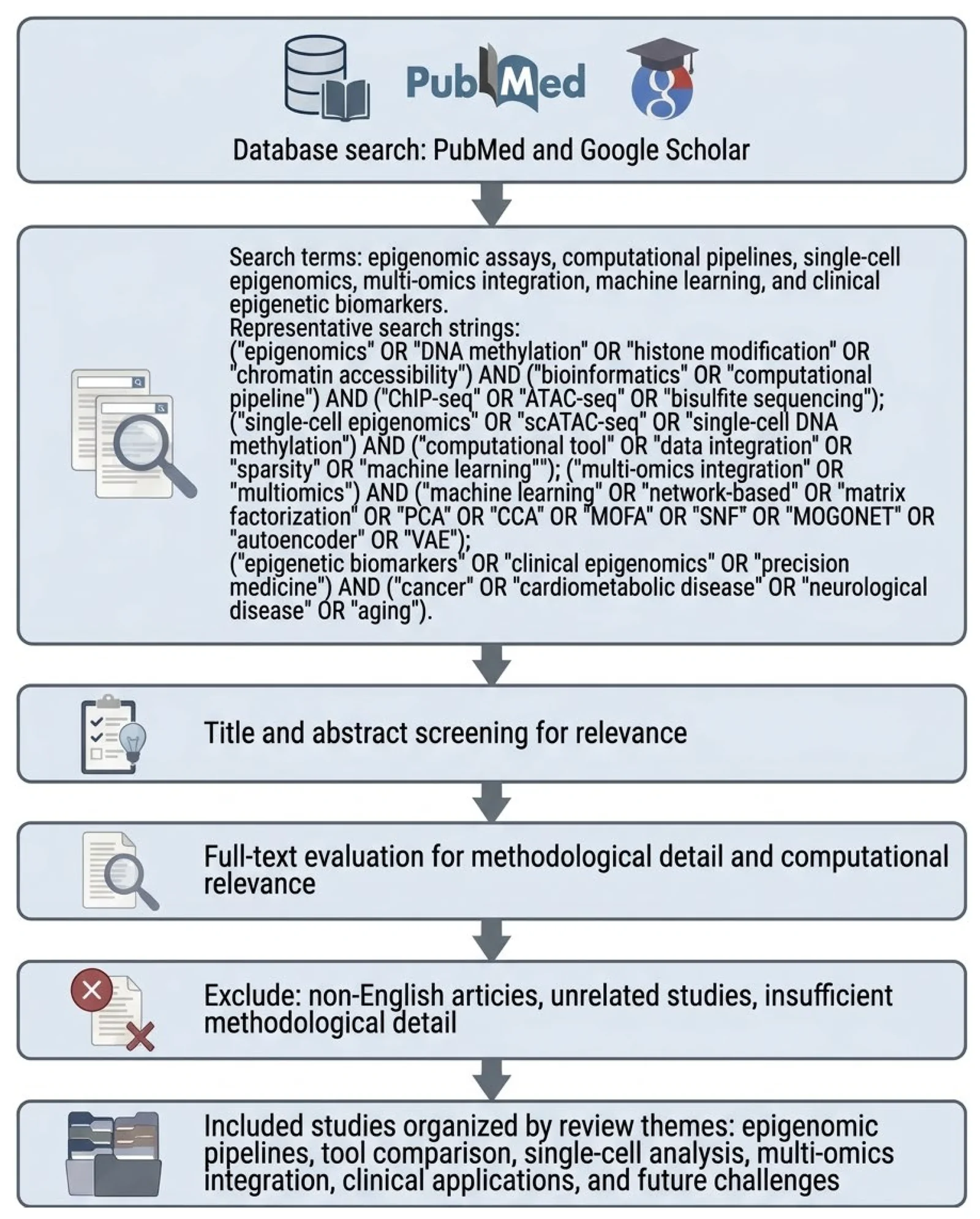
Literature selection workflow.

The selection of studies was restricted by relevance to the area of interest of this review. We prioritized peer-reviewed articles detailing popular experimental platforms, established and emerging computational tools, and integration systems and clinical uses in health and disease. Where needed, landmark studies were included to provide historical context, but more recent studies were prioritized to represent the current developments in the field. Since this is a narrative review, the literature selection was not intended to be exhaustive, and no PRISMA-style screening framework was utilized. The narrative-review literature selection workflow is summarized in Figure 1.

## 3. Epigenomic Data and Analysis Pipelines

Epigenomic data is typically stored in formats that can be indexed by genomic coordinates to enable integrative and comparative analysis. After processing, the information is represented as region sets or tracks mapped to reference genomes. This information is encoded in standard formats such as BED, bigWig, or narrowPeak files that indicate genomic regions containing specific epigenetic marks or quantitative data [13].

To simplify the handling of complex epigenomic datasets, especially in single-cell epigenomics, software packages like EpiScanpy (version 0.4.0) can process single-cell DNA methylation and chromatin accessibility data. These packages include tools for clustering, dimension reduction, and differential analysis specifically designed for epigenomic properties [14]. Furthermore, data integration initiatives, such as the VISION project, integrate various epigenomic marks and transcriptomic data to produce large-scale maps of regulatory landscapes. These maps are essential for annotating functional genomic elements [15]. Resources like the NCBI Epigenomics database have thousands of epigenomic datasets available in standard formats to enable streamlined data retrieval, visualization, and large-scale genomic analysis [16]. Additionally, programmatic interfaces such as the DeepBlue Epigenomic Data Server allow researchers to access, filter, and summarize data from large consortia, including ENCODE and Roadmap Epigenomics, thereby supporting automated and reproducible workflows [13]. Large-scale consortium efforts have fundamentally shaped the data landscape of integrative epigenomics. The ENCODE Consortium has systematically identified functional genomic elements, such as histone modifications, transcription factor binding sites, chromatin accessibility, DNA methylation, chromatin organization features, and RNA-associated signals, across diverse biological samples through standardized experimental protocols and uniform bioinformatics [17]. ENCODE-derived resources such as blacklist regions of anomalous signal are commonly used as preprocessing references for ChIP-seq, ATAC-seq, and other assays [18]. Complementing this, the Roadmap Epigenomics Project produced 111 reference human epigenomes and provided the chromatin-state annotation frameworks that researchers continue to use broadly to interpret genome-wide epigenetic regulation [19]. All these projects enabled the development of data standards, processing conventions, and reference epigenomes underpinning many computational tools, integrative frameworks, and benchmarking strategies used in epigenomics.

**Figure 2:**
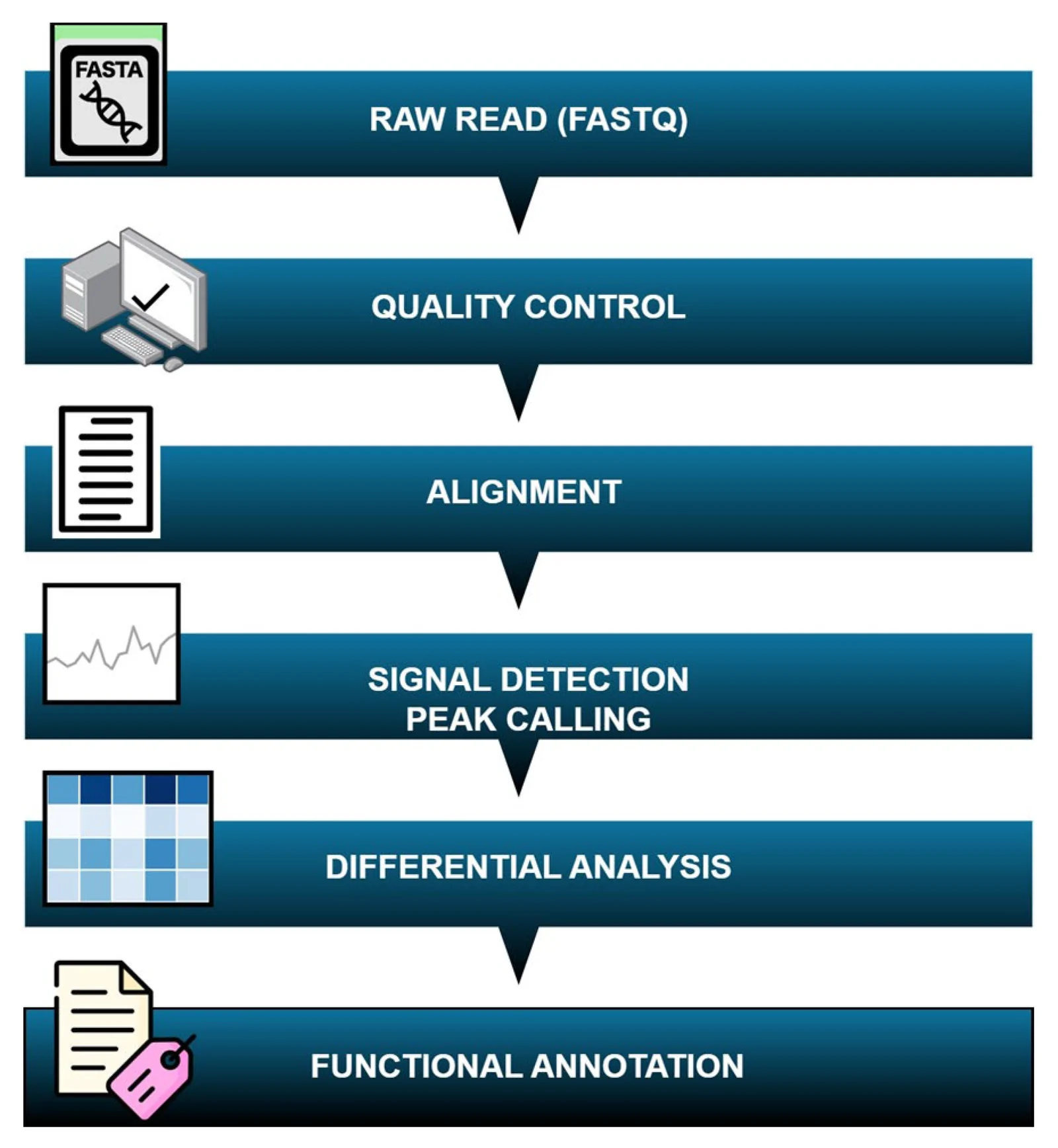
Standard computational pipeline for epigenomic data processing. Schematic representation of the bioinformatic workflow used to transform raw sequencing output into biological insights. The process follows a modular hierarchy from raw reads to functional annotation and final biological interpretation.

Although current experimental approaches to epigenomics include various assays such as DNA methylation profiling, ChIP-seq, and chromatin accessibility mapping, most studies follow a standardized computational workflow (Figure 2) to ensure consistency and reliability in data analysis. The workflow begins with a quality assessment of raw sequencing reads, usually in FASTQ format, to evaluate the quality and integrity of data produced by the high-throughput platforms. The filtration of low-quality reads, removal of adapter sequences, and elimination of potential contaminants are essential quality control steps to prevent errors in downstream analyses [20,21]. The interpretation of FastQC/MultiQC reports should be based on general sequencing-quality metrics as well as assay-specific QC checkpoints. When processing Illumina short-read epigenomic data, a standard rule of thumb is that at least 80% of bases should have a Phred quality score of Q30 or higher, which represents a base-call accuracy of 99.9%. Other metrics specific to each assay should also be considered, such as bisulfite conversion efficiency for WGBS/RRBS, FRiP score and signal-to-noise ratio for ChIP-seq, and TSS enrichment, fragment-size distribution, and mitochondrial read fraction for ATAC-seq [22,23,24,25].

Following quality control, the filtered reads are aligned to a reference genome using specialized alignment tools tailored to each assay as different assays require distinct aligners. Accurate alignment is critical because it determines the genomic locations from which the sequencing reads originate and forms the basis for signal detection. Once epigenetic signals are identified, differential analysis is performed to compare signals across different biological conditions (e.g., healthy versus diseased tissues) or experimental groups (e.g., treated versus control samples). Such comparisons can identify regions of the genome that exhibit significant epigenetic changes, which are often associated with regulatory functions or disease mechanisms. The final stage of the workflow is the functional annotation, where identified epigenetic regions are linked to genomic features such as genes, promoters, enhancers, or other regulatory features.

Every step of this workflow relies on specific bioinformatics tools chosen based on the assay type or research question. Errors or biases introduced in early stages, such as low-quality reads or misalignment, can propagate through the pipeline and lead to false biological findings. Therefore, pipeline-based decisions for epigenomic analysis are not only technical steps, but also critical factors in downstream inference that impact biological interpretation. For instance, normalization algorithms vary in how they handle sequencing depth and signal-to-noise ratios. Notably, the S3norm method was developed to normalize both parameters simultaneously and has been shown to capture biological variation more reliably than previous methods. S3norm simultaneously adjusts these parameters across datasets using a nonlinear transformation. Unlike methods that primarily correct either peak or background signals, S3norm is designed to preserve biological variation while reducing technical noise, making it useful for quantitative comparisons across samples and conditions [26]. Similarly, interpretations of the same datasets can vary depending on the functional annotation strategy used, as annotation reproducibility is often cell-type-dependent [27]. These challenges are further intensified in single-cell epigenomics, where the feature space construction and integration strategies significantly influence clustering, cell-type discrimination, and downstream inference [14].

Standardization and reproducibility remain critical issues in epigenomic analysis as technical and biological variation can significantly impact data quality and interpretation. The sequencing depth and signal representation can be altered by differences in preprocessing parameters, such as read filtering, duplicate removal, and adapter trimming. The sensitivity and specificity of identified features are also affected by parameters such as reference genome selection, sequencing depth, peak-calling algorithm and threshold settings. In ChIP-based experiments, antibody quality is a source of variability that cannot be addressed by computation. However, other technical factors such as batch effects in sample manipulation, library preparation, and sequencing conditions can further obscure the underlying biology. This issue is particularly acute in single-cell epigenomics, where low detection rates and sparse data matrices make results highly dependent on chosen strategies for feature construction, normalization, and integration [28,29].

Integrative frameworks like compEpiTools, methylPipe, and EpiScanpy can enhance harmonization by establishing shared feature spaces and annotation strategies. However, meaningful cross-platform integration still relies on deliberate normalization, batch correction, consensus reference annotations, and transparent parameter reporting [14]. Collectively, these challenges demonstrate that epigenomic pipelines are not universally applicable; analytical decisions at every stage affect the reproducibility, interpretability, and comparability of studies. Increased standardization, clear reporting of workflow parameters, and systematic benchmarking across assays and platforms are essential to facilitate sound biological inference and trustworthy clinical translation [29,30]. Thus, the design and implementation of standardized tool-selection workflows (Table 1) are essential to improve inter-study reproducibility and provide robust insights into epigenetic regulation and its implications for health and disease [19,31,32,33].

## 4. Comparative Evaluation of Epigenomics Tools

**Figure 3:**
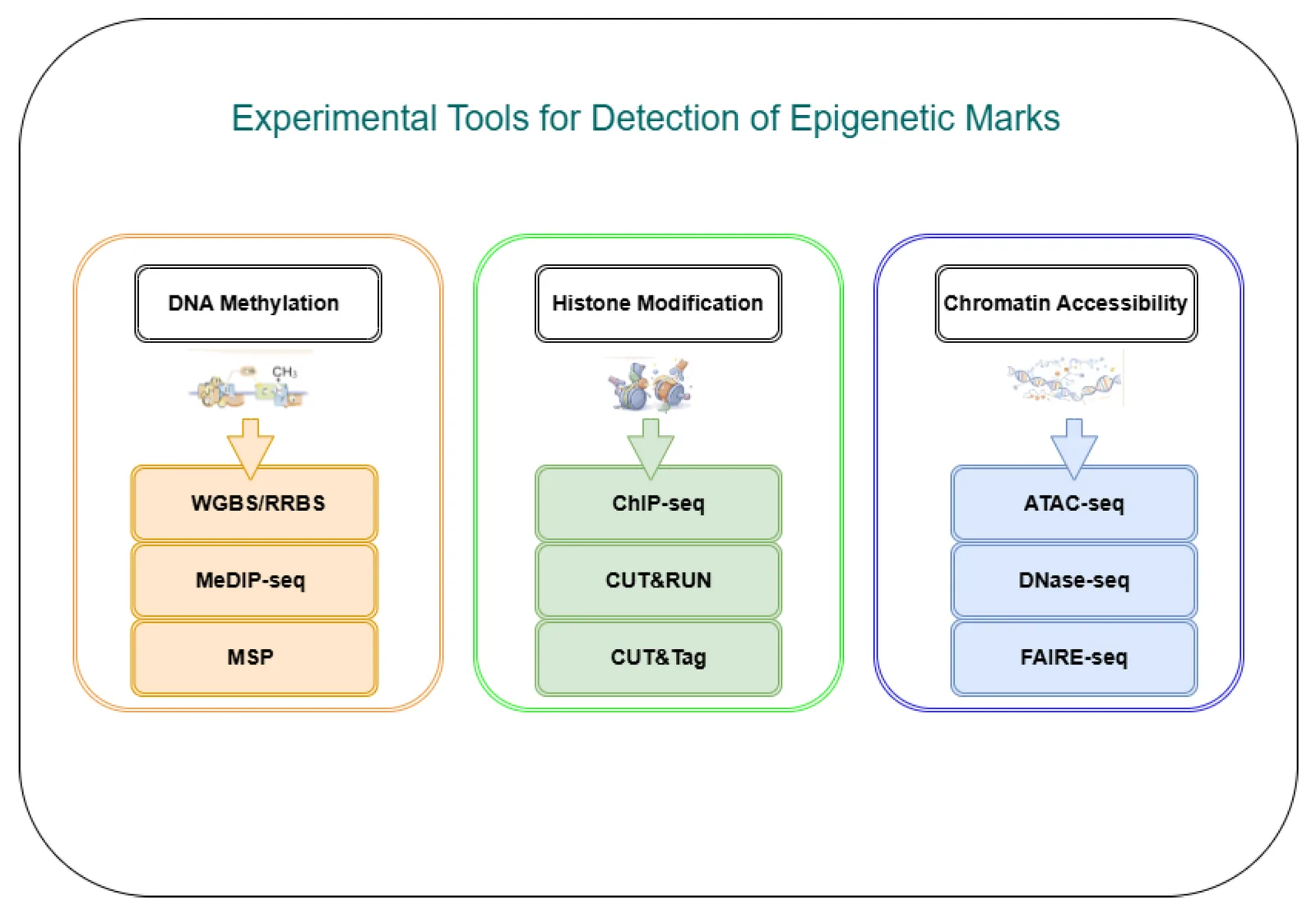
Experimental modalities for profiling epigenetic landscapes. A hierarchical classification of experimental methodologies used to detect and quantify various epigenetic modifications using wet lab. The figure categorizes tools into three primary biological domains.

Despite the availability of numerous wet lab epigenetic tools (Figure 3) for specific analytical stages, their performance remains dependent on the assay type, signal properties, and dataset size to be analyzed. Therefore, a meaningful comparison must not only focus on the major function of a tool but also consider its reproducibility, scalability, usability, downstream compatibility, and applicability to a specific experimental design. No single tool is universally superior; rather, choices depend largely on requirements such as sensitivity, interpretability, efficiency, and ease of integration into existing workflows.

### 4.1. Tools Based on Epigenetic Markers

For epigenomic analysis, two widely used tools for DNA methylation are Bismark, followed by MethylKit. WGBS (Whole Genome Bisulfite Sequencing) and RRBS (Reduced Representation Bisulfite Sequencing) DNA methylation profiling techniques can determine CpG methylation patterns genome-wide [9,34,35]. Additionally, MeDIP-seq (Methylated DNA Immunoprecipitation Sequencing) identifies methylated DNA sites using 5-methylcytosine antibodies [9,36,37]. MSP (Methylation-Specific Polymerase Chain Reaction) remains one of the oldest techniques for identifying the presence of DNA methylation at precise locations [38].

Besides the classical bisulfite-based technologies (WGBS, RRBS, and MSP/qMSP), new DNA methylation platforms are gaining increasing popularity for biomarker discovery and clinical translation. Illumina EPIC/EPIC v2 arrays are widely used in large cohort studies and epigenetic-clock research because they provide cost-effective profiling of predefined CpG sites [39,40]. Targeted bisulfite sequencing can be used to validate candidate methylation biomarkers at high depth, and cfDNA methylation profiling can be used for liquid biopsy, early cancer detection, tissue of origin prediction, and disease monitoring [41,42,43,44]. Direct detection of methylation in native DNA is possible using long-read nanopore or PacBio sequencing, and single-cell methylome sequencing can be used to study the methylation heterogeneity at the cellular level [45,46,47,48,49]. These platforms are compared in Table 2.

Long-read sequencing is also becoming available for direct methylation detection of native DNA. The base modifications are detected by changes in ionic current by Oxford Nanopore sequencing, while PacBio SMRT/HiFi sequencing infers modified bases by changes in polymerase kinetic signals [53,54]. These platforms can address methylation in repetitive regions, structural variants, haplotypes, and allele-specific contexts that are hard to analyze using short-read bisulfite methods [45]. However, accuracy depends on sequencing depth, chemistry, and base-modification calling algorithms, requiring careful computational validation [55].

Bismark is a methylation caller specifically designed for bisulfite sequencing that offers flexible alignment. It efficiently maps sequencing reads generated from bisulfite-treated DNA and performs methylation calling in a single step. This tool allows researchers to distinguish cytosine methylation across different sequence contexts such as CpG, CHG, and CHH and can provide detailed methylation profiles at single-base resolution. This type of functionality allows the visualization and interpretation of methylation throughout the genome. Furthermore, Bismark is open source, freely available, and supports time-efficient processing, making it widely accessible for many laboratories [56].

However, Bismark has limitations, as it is heavily dependent on the quality of the data generated from bisulfite-treated DNA. Aligning large bisulfite sequencing datasets requires a significant amount of processing time and resources for whole-genome analyses. Bismark is popular due to its alignment accuracy and identification capabilities; however, it can be expensive to use for whole-genome datasets [56]. In comparison, BS-Seeker2 may provide faster alignment in some computational settings, although Bismark remains more widely adopted in standard workflows (Table 1). Studies show that Bismark is a reliable option for stable bisulfite-seq alignment and integrated methylation calling. On the other hand, BS-Seeker2 might lower runtime and memory use in certain situations (Table 3).

To perform downstream differential methylation analysis, methylKit offers a powerful statistical framework and experimental flexibility. However, it has limitations, such as the fact its performance can be affected by low sequencing depth and coverage in sensitive regions. Consequently, selecting tools for methylation analysis often involves a trade-off between accuracy of alignment, runtime, ease of adoption, statistical flexibility, and dataset size.

#### 4.1.1. Chromatin and Histone Analysis

Histone modification mapping is typically performed using ChIP-seq and more recent enzyme-tethering methods like CUT&RUN and CUT&Tag [10,57,58]. Numerous computational tools exist for ChIP-seq data, focusing on data processing, peak calling, normalization, visualization, and downstream analysis. In the case of ChIP-seq data analysis, tools like PeakSeq (v1.3) and DROMPA (v1.20.5) (Discrepancy Restricted Overlapping Mapped Peak Analyzer) are used for peak calling. These tools identify and score protein-binding regions on DNA by comparing experimental samples against control samples. PeakSeq uses a 2-step method for sequence mapping to control open chromatin bias, which improves experimental design and sequencing depth estimation [59,60].

DROMPA is designed for efficient peak calling and visualization, particularly for identifying binding profiles in repetitive sequences and handling both broad and sharp peaks. Moreover, DROMPA is user-friendly and accessible to users with a limited bioinformatics background [61]. In chromatin profiling, signal shape and specific analysis objectives are critical factors in selecting the most appropriate tool. PeakSeq offers improved signal detection by correcting background noise. Moreover, it is valuable when there is a concern about open-chromatin bias. DROMPA is efficient at identifying and visualizing peaks, making it especially useful for studying both broad and sharp peak profiles within a user-friendly framework.

Tools like CoBRA (Combined Bisulfite Restriction Analysis) offer organized bioinformatics workflows for analyzing ChIP-seq and ATAC-seq data. These tools streamline several critical steps, including normalization, copy number variation correction, clustering, differential peak calling, motifs identification, and pathways analysis. By integrating these processes, researchers can better understand chromatin accessibility and the complexities of protein-DNA interactions [62].

Other resources, such as EaSeq offer interactive environments that combine genome browsing with user-friendly tools for data exploration, abstraction, and visualization. This transparency allows researchers to generate hypotheses and analyze large datasets in a reproducible manner [63]. Ultimately, a systematic evaluation of over 30 computational tools for differential ChIP-seq analysis indicates that tool performance depends significantly on the size and shape of peaks as well as specific biological contexts. This emphasizes the need for tailored selection of algorithms based on the experimental design and biological question to maximize both accuracy and usability [60,64]. Comparative benchmarking indicates that there is no universal ChIP-seq analysis tool that is best at all peak structures and biological contexts. Instead, the selection of tools should be made based on the peak width, signal-to-noise profile, visualization requirements, and intended downstream analysis.

#### 4.1.2. Assay-Specific and Advanced Methods

Advanced methods like MAPS (Model-based Analysis of PLAC seq) have been developed for integrated analyses combining ChIP with chromatin conformation approaches (e.g., PLAC-seq, HiChIP). By applying statistical modeling, MAPS identifies long-range chromatin interactions anchored at protein-bound sites, significantly expanding the insights gained from standard ChIP-based assays [65].

Recent advancements have introduced assay-specific tools that further enhance chromatin profiling workflows. SEACR (Sparse Enrichment Analysis for CUT&RUN) serves as a selective peak caller for CUT&RUN data [66] and GoPeaks is designed as a selective peak caller for CUT&Tag histone-modification profiling [67]. LanceOtron, a machine learning-driven peak caller that is designed to address limitations of traditional methods, has been used in recent benchmarking studies to compare the performance across diverse chromatin profiling datasets [68]. These more recent tools point to the ongoing shift toward assay-specific and learning-based methods of detecting peaks. For example, Omnipeak was recently developed to call chromatin-mark peaks on both narrow and broad signal profiles, with a focus on peak stability and reproducibility. These findings highlight the significance of tool selection based on peak architecture and experimental design [69]. The choice of tool should account for factors such as data characteristics, biological questions, computational resources, and user expertise to effectively interpret protein-DNA interaction landscapes [59,61,64]. In practice, assay-specific tool selection should be based on the type of histone marks, the structure of the peaks, and the goals for the downstream applications rather than the assumption that one tool for peak calling is ideal for all chromatin profiling applications.

#### 4.1.3. ATAC-Seq and Regulatory Analysis

ATAC-seq is an important technique for identifying regulatory regions, including enhancers and promoters. The rapid adoption of ATAC-seq has been accompanied by the development of specialized computational tools tailored to process, analyze, and interpret ATAC-seq data effectively. MACS2, originally developed for ChIP-seq peak calling, has been adapted for ATAC-seq to identify regions of accessible chromatin by detecting significant enrichment in sequencing reads [10,11]. It remains a robust and widely used peak caller for ATAC-seq data, being particularly effective when enrichment profiles are relatively sharp [70,71]. HOMER (Hypergeometric Optimization of Motif EnRichment) is frequently applied after peak calling to perform motif discovery within accessible chromatin regions. It allows researchers to infer which transcription factors and other regulatory proteins may bind to open chromatin sites [71].

GenomicRanges (v1.64.0) is a software tool for region-based comparisons, enabling analysis of identified peaks in the context of genomic features, overlap queries, and integration of ATAC-seq peaks with other genomic datasets. This enhances the ability to compare chromatin accessibility across different conditions or cell types [71].

FAIRE-seq is a specialized technique for determining open chromatin regions by isolating the nucleosome-depleted DNA [72]. Complementary tools, such as ATACgraph provide ATAC-seq-specific functionalities, including quality control, differential accessibility analysis, and peak identification of both nucleosome-free and nucleosome-occupied regions. Designed with user-friendly interfaces, these tools are particularly accessible to bench scientists with limited bioinformatics training [73].

HMMRATAC uses a Hidden Markov Model to find accessible regions within the genome. Unlike standard methods, it can accurately distinguish between nucleosome-free DNA and nucleosome-occupied DNA throughout the genome. This method is more sensitive than conventional peak calling approaches [74]. Comprehensive pipelines such as CoBRA support reproducible and scalable workflows for both ChIP-seq and ATAC-seq analysis. These pipelines integrate several critical analytical stages, including normalization, differential peak calling, motif enrichment, and pathway analysis to efficiently interpret chromatin accessibility profiles [62].

These tools allow researchers to map the regulatory framework, such as the active enhancers, promoters, and binding sites of transcription factors, which further helps to understand gene regulation and cellular identity [71,75]. Selected core tools used in these workflows are summarized in Table 1. The selection of an ATAC-seq workflow is not universal; rather, it should be tailored to the specific biological question and the expected profile of the chromatin signal. MACS2 is a widely used and effective general-purpose peak caller, especially for accessible chromatin regions with sharp enrichment profiles. However, HMMRATAC can be beneficial when it is necessary to distinguish between nucleosome-free and nucleosome-bound regions. HOMER is then the preferred choice for discovering downstream motifs, whereas GenomicRanges is useful when comparative and integrative analyses across conditions or datasets are required. Ultimately, ATAC-seq workflows are more reliable when selected based on the specific requirements for peak detection sensitivity, chromatin-state resolution, motif analysis, or cross-dataset comparisons [70,71,74]. Representative benchmark studies comparing selected epigenomic analysis tools are summarized in Table 4.

### 4.2. Tools for Functional Annotation and Data Interpretation

To interpret the biological significance of epigenomic signals, researchers employ functional annotation and integration tools that connect these signals to specific genomic features and regulatory functions. BEDTools is an effective and highly versatile program that can be used to compare genomic features (epigenomic peaks), allowing for the annotation of regions in BED or GFF formats. This enables researchers to determine the overlap between the epigenetic marks and the known genomic elements [76]. ChIPseeker is an R/Bioconductor package that is specifically used to annotate ChIP-seq or other epigenomic peaks with nearest genes and genomic features such as promoters, exons, and introns. Beyond basic annotation, it offers advanced capabilities including peak comparison and visualization around the transcription start sites. By leveraging thousands of publicly available datasets, ChIPseeker assists in identifying co-regulation patterns and transcription factor complexes [77,78]. GREAT (Genomic Regions Enrichment of Annotations Tool) enhances the interpretation of cis-regulatory region functions by incorporating both the proximal and distal regulatory regions of genes [79]. Unlike tools that rely solely on “nearest-gene” relationships, GREAT employs sophisticated statistical models to assess enrichment across various ontologies. This provides researchers with deeper insights into the biological processes and pathways associated with specific epigenomic regions.

GREAT is available as a web application and designed to manage false positive results in mapping of genome-wide data sets and is useful not only to ChIP-seq but can also be applied to open chromatin and other types of epigenomic data [79]. These tools allow extensive linking of epigenetic alterations to gene regulation and biological pathways, allowing researchers to gain valuable functional information from raw epigenomic data [76,77,79]. Together with tools like BEDTools and ChIPseeker, GREAT enables researchers to extract valuable functional information from raw epigenomic data, providing a clearer picture of how the epigenome influences cellular behavior.

Functional annotation tools are required for interpreting epigenomic regions, yet their utility depends heavily on the specific annotation approach used. While overlap-based methods like BEDTools are versatile and effective for intersecting peaks with known genomic features, they often fail to identify which specific genes or regulatory programs are biologically affected [76]. ChIPseeker readily performs the nearest-gene annotation and peak distribution analysis, but nearest-gene assignment can be ambiguous for distal enhancers, intergenic peaks, or loci where multiple potential targets exist [77,78]. GREAT partially overcomes this shortcoming by including both proximal and distal regulatory domains, although its interpretations remain dependent on pre-defined association rules and may yield false positives when regulatory regions are indirectly associated with genes [79].

These limitations are particularly significant because most epigenomic signals reside in non-coding regions. In these areas, functional implications are often context-dependent and hard to understand without integrating supplementary data such as chromatin conformation data, expression correlation data, or multi-omics integration datasets. Consequently, annotation results must be interpreted with caution, and tools should be selected based on the specific research objective—whether that is a rapid genomic overlap, a gene-focused annotation, or a broad regulatory analysis.

### 4.3. Tools for Single-Cell Epigenomic Technologies

**Figure 4:**
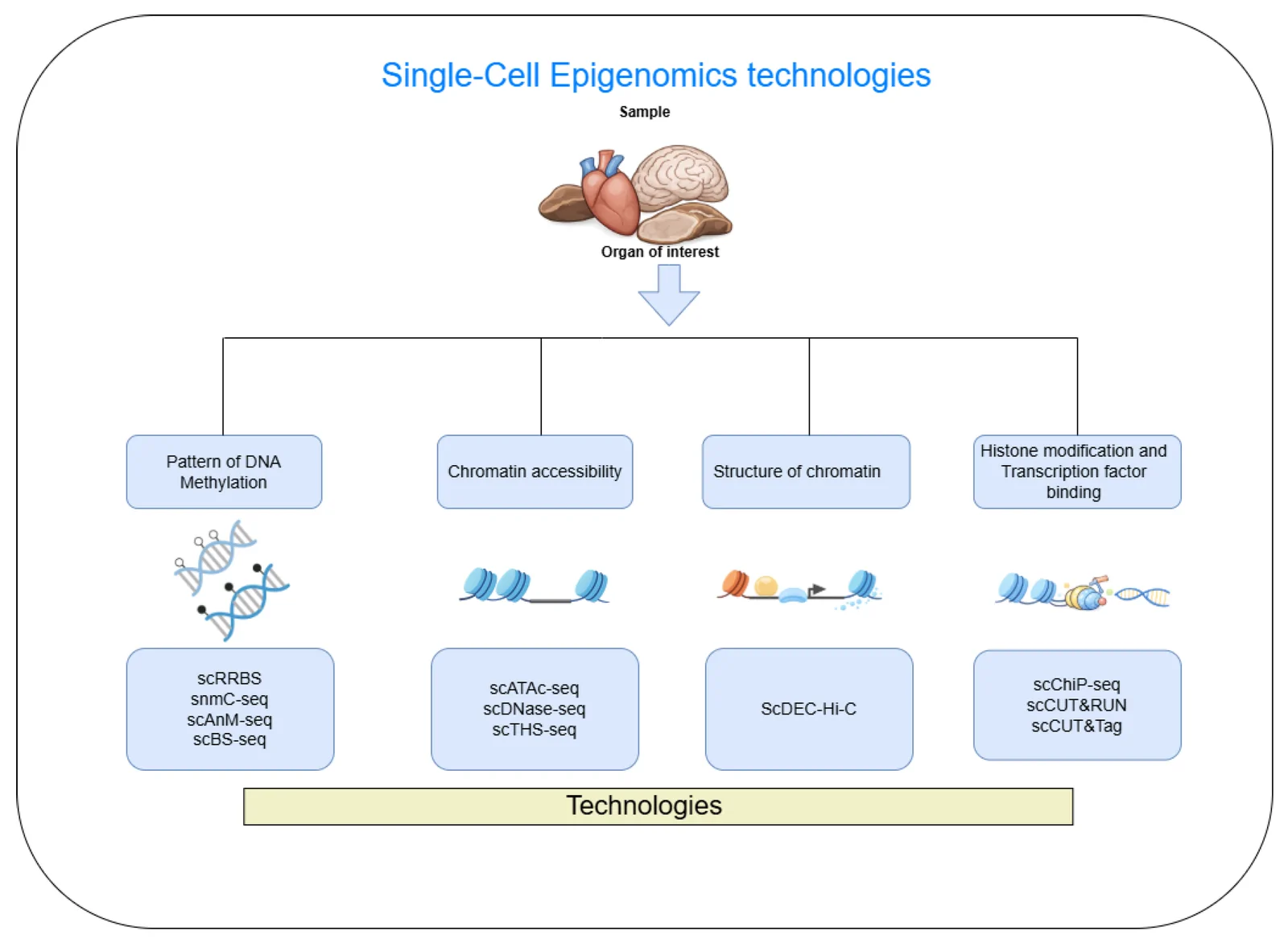
Illustration of single-cell epigenomics technologies. Schematic workflow for the application of single-cell sequencing technologies across various epigenetic layers. Tissues are dissociated to enable the profiling of individual cells, bypassing the averaging effect of bulk sequencing. These technologies are categorized into four primary functional domains.

Recent advancements in sequencing technologies have moved epigenomic profiling beyond bulk analysis, where signal averaging can obscure critical cellular heterogeneity across large cell populations. Single-cell epigenomics provides a high-resolution view of regulatory dynamics, capturing cell-to-cell variations in DNA methylation, chromatin accessibility, histone modifications, and chromosomal conformation. These methods (Figure 4) are essential for understanding cellular identity, plasticity, and tissue-specific functions in complex biological systems.

#### 4.3.1. Computational Frameworks for Profiling Epigenetic Features at Single-Cell Resolution

scATAC-seq and single-cell DNA methylation sequencing allow researchers to identify regulatory states in individual cells. ArchR, Signac, and SnapATAC are advanced computational tools developed to support key analytical tasks in single-cell chromatin accessibility studies. These platforms support essential analytical tasks, including peak calling, dimensionality reduction, cell clustering, and integration with single-cell transcriptomic data [80,81,82]. A variety of specialized assays allow for the detailed mapping of individual epigenetic features of each cell, such as DNA methylation, chromatin accessibility, histone modifications and chromosomal conformations.

The pattern of DNA methylation in single cells can be identified by the scWGBS (single-cell whole genome bisulfite sequencing), single-cell reduced representation bisulfite sequencing (scRRBS), single-cell bisulfite sequencing (scBS-seq), and single-nucleus methylcytosine sequencing (snmC-seq). This capability permits the investigation of heterogeneity in DNA methylation on an individual cell basis, providing deeper insight into cell-to-cell epigenetic variation. This approach facilitates a deeper understanding of dynamic changes in cell states during biological processes such as differentiation as well as in disease contexts, such as cancer [47]. Chromatin accessibility profiling can be performed with assays like single-cell ATAC-seq (scATAC-seq), scDNase-seq, and scTHS-seq, that define the nucleosome-depleted regions and regulatory elements like promoters and enhancers. Histone modifications and transcription factor binding can be analyzed by using single-cell ChIP-seq, CUT&RUN and CUT&Tag, which enable the mapping of proteins and regulatory complexes associated with the chromatin in individual cells. ScDEC-Hi-C (single-cell deep embedded clustering) can be used to analyze the structure of chromatin and long-range genomic interactions in three-dimensional architecture by measuring spatial proximity between DNA loci [83,84,85].

#### 4.3.2. Emerging Frontiers: Spatial and Genomic Amplification

For complex tissues, spatially resolved single-cell epigenomics has emerged to link gene regulation with physical location. For instance, in situ segmentation and multiplexed imaging enable the profiling of histone modifications and enhancer activity in individual cells across tissue sections, thus linking spatial gene regulation with developmental and functional biology [73]. Furthermore, analyzing the limited DNA found in single cells is made possible through whole-genome amplification techniques like MALBAC (Multiple Annealing and Looping-Based Amplification Cycles) or LIANTI (Linear Amplification via Transposon Insertion). These methods ensure reliable genomic profiling even from extremely small quantities of starting material.

#### 4.3.3. Challenges in Single-Cell Analysis

However, single-cell epigenomics data analysis is subject to several limitations, primarily including high technical noise, sparsity of data matrices, and increased computational complexity. Firstly, it is often challenging to distinguish true biological signals from artifacts due to high technical noise in single-cell epigenomic data. Addressing this requires specialized noise models and robust computational frameworks to improve signal-to-noise ratio and reliability [86]. Secondly, data sparsity is a hallmark of single-cell epigenomics. For instance, in scATAC-seq data, around 1% to 10% of chromatin accessibility peaks are detected per cell due to limited sequencing depth and DNA sampling from diploid genomes. These highly sparse matrices often hinder essential downstream tasks such as cell-type identification and clustering [87]. As a benchmark example, Chen et al. evaluated 10 scATAC-seq computational methods across 13 synthetic and real datasets. Their findings showed that data sparsity, sequencing coverage, technical noise, feature construction, and clustering performance are all critical parameters in single-cell chromatin accessibility analysis [87]. Finally, the high dimensionality and large scale of single-cell epigenomic datasets result in increased computational complexity. Processing such large-scale, noisy data requires advanced algorithms, including deep learning, graph neural networks, and variational autoencoders. Moreover, they must reduce data size, correct batch effects, and interpret biological data efficiently. Despite recent advances, the complexity of these analyses remains a bottleneck, especially for the integration of multimodal single-cell omics datasets [86,88,89]. The most relevant machine learning frameworks to this challenge are discussed in Section 5.4; further, the current challenges and future directions are considered in Section 7.

#### 4.3.4. Specialized Single-Cell Analysis Tools

Researchers have developed specialized single-cell epigenomic tools to address two major challenges: sparse chromatin-accessibility matrices and genetic variation interpretation in cell-type-specific regulatory contexts. Tools such as scCASE and CASTLE are designed to improve the analysis of sparse scATAC-seq data. scCASE enhances chromatin-accessibility signals and mitigates dropout-related sparsity, whereas CASTLE performs latent-space embedding to recover meaningful regulatory structure from sparse single-cell data [90,91]. In contrast, SCAVENGE serves for a different purpose: linking disease- and trait-associated genetic variants to regulatory cell states. SCAVENGE combines GWAS or fine-mapped variants with single-cell chromatin-accessibility data by constructing a cell-similarity network, identifying variant-enriched “seed” cells, and propagating the enrichment signal across the network to prioritize cell states associated with the genetic signal [80]. This approach differs from standard cluster-level enrichment because it can identify variant-associated signals at higher single-cell resolution and may help detect rare or transitional cell states. scCASE and CASTLE are appropriate when the primary challenge is sparsity, signal recovery, or latent representation of scATAC-seq data. SCAVENGE is useful when the research question involves connecting GWAS or fine-mapped variants to regulatory cell states. Thus, specialized single-cell epigenomic tools are advancing the field beyond basic clustering toward more targeted analyses of signal enhancement, genetic interpretation, and regulatory-state discovery [87,90,91,92]. The implications of this sparsity for cross-modal multi-omics integration are discussed further in Section 5.5.

## 5. Integration of Epigenomics with Other Omics Data

**Figure 5:**
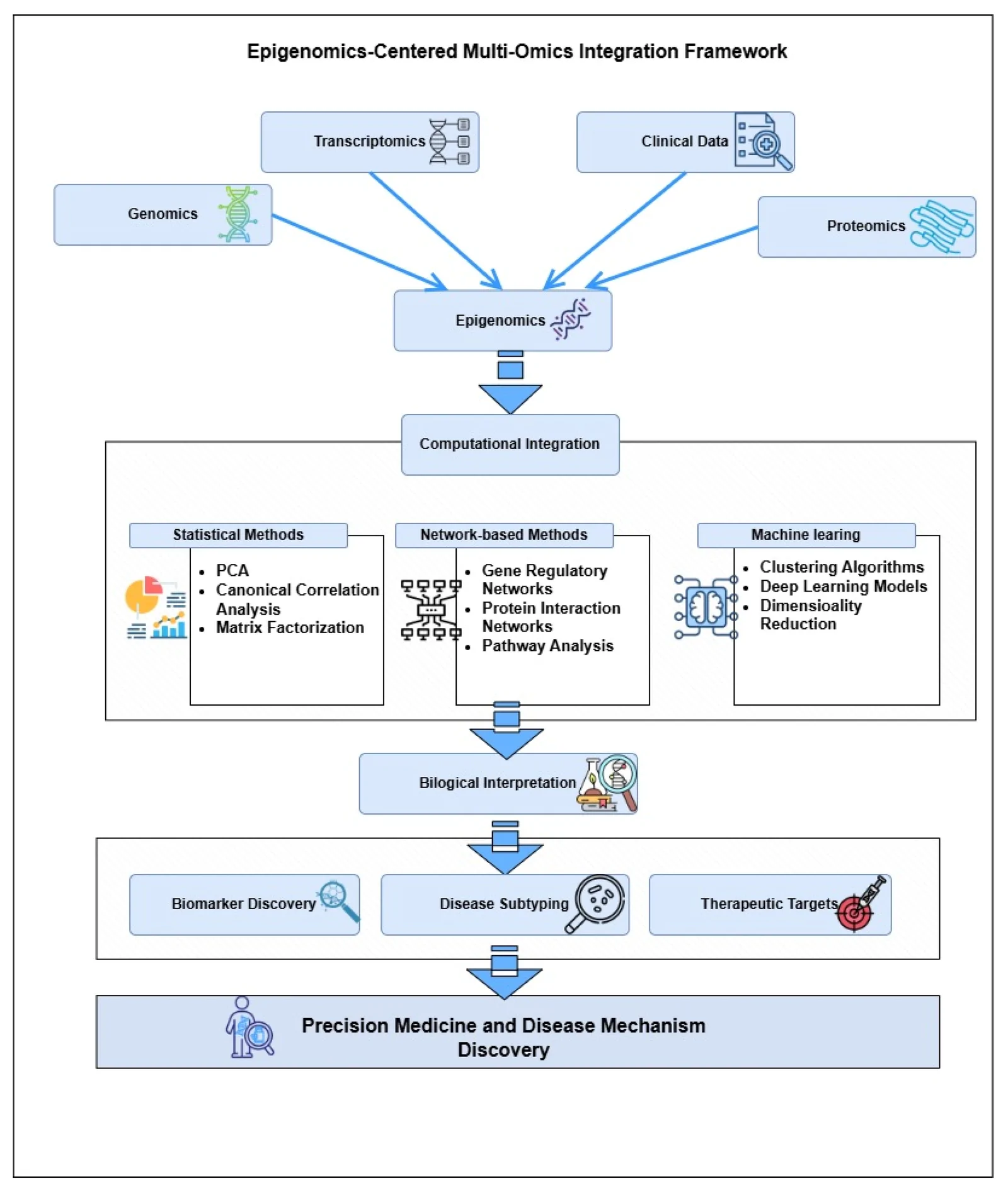
Representation of the multi-omics integration framework. Strategies enabling complex analysis of biological systems through the integration of multiple molecular datasets. A conceptual workflow demonstrates the convergence of diverse biological data streams through computational intelligence to drive clinical discovery. The process is divided into data acquisition, computational integration, biological interpretation and clinical applications.

Human health and disease are too biologically complex to be entirely represented by any single omics modality. The integration of epigenomics with genomics, transcriptomics, proteomics, radiomics and clinical data helps to understand more complex biological insights through the integration of multiple molecular data. The most common strategies of computational integration of heterogeneous datasets are statistics-based approaches principal component analysis (PCA) or canonical correlation analysis (CCA), network-based approaches (such as gene regulatory and protein interaction networks), and machine learning algorithms (like clustering algorithms or deep learning models). These analytical tools facilitate the identification of disease-associated molecular signatures, thereby enabling biomarker discovery, disease subtyping, and identification of therapeutic targets (Figure 5).

The selection of a specific strategy is usually based on biological questions, like data type (e.g., bulk or single cell), and the required level of interpretability. Ultimately, method selection (Table 3) must balance predictive performance with scalability, robustness, and the ability to harmonize data across platforms [93,94,95,96]. To provide more evidence-based guidance for tool selection, representative benchmark studies across major epigenomic and multi-omics workflow categories are summarized in Table 4.

**Table 3:** Summary of integrative omics tools and computational strategies. This table provides a comparative overview of key computational tools used for the integration of epigenomics with other omics modalities. It details the underlying mathematical strategies, the types of datasets typically employed, key performance findings, and the specific research contexts where each tool is effective.

Tool Name	Integration Strategy	Dataset Used	Key Findings	Best Used When	Interpretability/Trade-Off
MOGONET	Graph convolutional networks/supervised deep learning	mRNA expression, DNA methylation, and miRNA expression across biomedical classification datasets.	Showed strong performance compared with other supervised multi-omics classification methods in the reported datasets [97].	Supervised classification is the main goal, and the dataset contains multiple omics layers with matched samples.	Strong predictive performance, but lower interpretability than factor-based methods and greater computational complexity.
MOFA/MOFA+	Matrix factorization/factor analysis	Simulated datasets and TCGA multi-omics benchmarks	MOFA/MOFA+ can rediscover common structures in benchmark datasets [98].	Unsupervised exploration of heterogeneous bulk multi-omics datasets; identifying shared vs. modality-specific variation	High interpretability and useful latent-factor structure, but may be less effective than nonlinear methods when interactions are highly complex
SNF	Network-based similarity fusion	Simulated CRC multi-omics and TCGA colorectal cancer datasets including DNA methylation, gene expression, and protein expression	Showed strong subtype-classification performance in the colorectal cancer benchmark study [99].	Patient stratification, subtype discovery, and similarity-based clustering across multiple modalities	Good for relationship-centered analysis, but less directly interpretable at the individual feature level
VAE	Deep learning/variational autoencoders	Single-cell parallel multi-omics datasets such as scRNA-seq + scATAC-seq and CITE-seq	Single cell multi-omics benchmarks suggest that the performance depends on tasks and metrics, so deep VAE variants are one of the widely studied but no overall numeric advantage is demonstrated in the provided passages [38,100,101].	Large, nonlinear, multimodal datasets where denoising, latent representation learning, or high-dimensional prediction is prioritized	Flexible and powerful for nonlinear modeling, but usually less interpretable and more computationally demanding
iCluster	Joint latent variable/matrix factorization	Simulations and TCGA breast cancer datasets	Able to recover shared structure with high accuracy in simulations, as well as generated competing clusterings on TCGA breast cancer data [98].	Integrative clustering and subtype discovery when interpretable latent structure is desired	More interpretable than deep learning approaches, but limited by stronger modeling assumptions
DIABLO (mixOmics)	Sparse PLS/supervised multi-omics integration	Multi-omics classification and biomarker discovery studies across matched cohorts	Supports correlated multi-omics feature selection and supervised discrimination across conditions [94].	Biomarker and supervised classification when interpretable multi-omics signatures are needed	High interpretability and feature selection strength, but less suited for purely unsupervised structure discovery
WGCNA	Network/module-based integration	Transcriptomic datasets with epigenetic overlays such as accessibility or methylation-associated analyses	Useful for identifying co-regulated modules and relating them to epigenetic changes [102].	Linking chromatin accessibility or methylation shifts to coordinated gene-expression modules	Strong biological interpretability at the module level, but not designed for complex multimodal prediction
PANDA/NetBID	Regulatory-network inference	Multi-omics regulatory studies integrating transcriptomic and epigenetic signals	Useful for identifying candidate master regulators and hidden drivers from regulatory-network structure [103,104].	Regulatory-network inference, transcription factor activity analysis, and mechanistic prioritization	High mechanistic value, but dependent on network assumptions and prior regulatory information
Seurat/Signac	Weighted nearest neighbor (WNN) analysis	Paired single-cell multi-omics such as 10x Multiome	Widely used for joint representation of RNA and chromatin accessibility in the same cells [82,105].	Paired single-cell RNA-seq and ATAC-seq integration, joint clustering, and peak-to-gene linkage	Highly practical and widely adopted, but mainly tailored to paired single-cell data
LIGER	Integrative non-negative matrix factorization (iNMF)	Unpaired or batch-affected single-cell multi-omics datasets	Effective for aligning datasets not measured in the same cells and for mitigating strong batch effects [106]	Unpaired single-cell integration or datasets generated across different platforms/labs	Useful for harmonization, though interpretation may be less direct than simpler factor-based bulk methods
OmiVAE/related autoencoder frameworks	Deep learning/autoencoder-based integration	Large cohort-level multi-omics datasets such as TCGA-style studies	Useful for latent compression and prediction in high-dimensional multi-omics settings [107]	Survival prediction, subtype prediction, or other nonlinear cohort-scale predictive tasks	Higher predictive flexibility, but reduced feature-level interpretability

**Table 4:** Representative benchmark studies comparing selected epigenomic and multi-omics tool categories. This table provides a structured summary of representative published benchmark evidence for key tool categories reviewed in Section 4.1, Section 4.2, Section 4.3 and Section 5. Performance values are approximate ranges; actual results depend on assay type, data quality, sequencing depth, genome size, and compute environment. For unsupervised integration methods, AUC is not the primary benchmark metric; clustering quality is more commonly assessed using measures such as NMI and silhouette score. GPU acceleration is often recommended for deep learning-based methods. Memory estimates assume large-cohort or whole-genome-scale inputs.

Tools Compared	Metrics Reported	Main Benchmark Finding	Practical Takeaway
Bismark, BS-Seeker2	Mapping rate (Bismark ≥ 95%), methylation calling accuracy (>99% for Bismark), runtime (BS-Seeker2 ~1.5–2× faster than Bismark), memory usage (Bismark 8–16 GB; BS-Seeker2 4–8 GB) [56,108].	Bismark remains a widely used standard for bisulfite-seq alignment and methylation calling, whereas BS-Seeker2 may provide faster alignment in some computational settings with comparable mapping utility.	Bismark is suitable when workflow stability and community-standard implementation are priorities, whereas BS-Seeker2 may be preferred when computational efficiency is more important.
MACS2, SICER	Sensitivity (MACS2 ~70–90%; SICER ~80–90% for broad marks), specificity (MACS2 > 90%; SICER ~75–85%), runtime (typically < 1 h for MACS2), memory usage (both usually ~2–4 GB) [109,110]	MACS2 performs well as a general-purpose peak caller, particularly for relatively sharp enrichment profiles, whereas SICER is more suitable for broad histone-mark regions.	MACS2 is generally preferred for narrow peaks and standard workflows; SICER is more useful for broad histone marks.
DiffBind and related DESeq2/edgeR-based workflows	Sensitivity and specificity reported as high in benchmark comparisons; runtime typically moderate (hours for large datasets); memory usage may exceed 16 GB for large DiffBind analyses [64].	Differential ChIP-seq tool performance varies substantially depending on peak width, data quality, and experimental design. No single framework is optimal across all benchmark settings.	Differential analysis tools should be selected according to peak architecture, replicate structure, and downstream objective, rather than by using a default method alone.
ArchR, Signac, SnapATAC	Cell-type identification accuracy reported as high for ArchR and Signac; runtime in hours for large datasets; memory usage approximately 16–32 GB for Signac and ≥32 GB recommended for ArchR; SnapATAC generally has moderate memory (~8–16 GB) [111,112]	All three tools are useful, but they differ in scalability, integration flexibility, and computational burden. ArchR and Signac perform strongly for integrative analyses, whereas SnapATAC remains useful for large-scale accessibility-focused workflows.	ArchR is useful for scalable scATAC-seq analysis, Signac is advantageous when scATAC-seq must be integrated with scRNA-seq, and SnapATAC is useful for large accessibility datasets when workflow complexity is manageable.
MOGONET vs. other supervised multi-omics classifiers	Classification accuracy/AUC > 0.90 on TCGA-style benchmark datasets; runtime in hours; memory usage typically 16–64 GB [97].	MOGONET outperformed several supervised multi-omics classification methods across biomedical datasets using mRNA, DNA methylation, and miRNA data.	MOGONET is appropriate when the main goal is supervised classification using matched multi-omics datasets, although its interpretability is lower than that of factor-based models.
SNF, iCluster, MOFA/MOFA+	Clustering accuracy, normalized mutual information (NMI), silhouette score, runtime, memory usage [99,113]	Benchmark studies indicate that performance depends strongly on data structure and analytical goal. SNF performs strongly for relationship-centered clustering, whereas MOFA/MOFA+ and iCluster are valuable when interpretable latent structure is desired.	SNF is useful for subtype discovery and similarity-based clustering, whereas MOFA/MOFA+ and iCluster are preferable when interpretability and latent-factor structure are priorities.
VAE-based methods, OmiVAE-related frameworks	Task-specific classification performance reported; OmiVAE-related frameworks show AUC ~0.80–0.92 in TCGA-style studies; runtime in hours with GPU preferred; memory usage typically 32–64 GB [100,101,107]	Widely used for nonlinear integration and latent space learning, but benchmark studies show that performance remains task-dependent and not uniformly superior across all datasets.	These Frameworks are useful when the main goal is nonlinear modelling, denoising, or high-dimensional prediction, but they usually require higher computational resources and offer less direct interpretability.

### 5.1. Statistical Methods

PCA and CCA are fundamental tools that allow the number of dimensions to be reduced and the relationship between modalities to be determined. PCA removes noise and redundancy by converting the original features into principal components, enabling further classification or regression frameworks. For instance, the combination of nonnegative PCA with support vector machines (NPCA-SVM) [114] has proven robust in identifying cancer biomarkers in microarray data. CCA determines the relationship between two or more types of omics data, can be combined with machine learning to extract canonical variables. These variables serve as an integrated and compact representation of shared biological signals across datasets [115,116]. Advanced variations, such as L2,1-norm-constrained CCA, boost the feature selection and noise resistance, which allows more discriminative and compact data representation in machine learning applications [117]. Nonlinear association discovery and feature extraction of high dimensional datasets are made possible through kernel and sparse implementations of CCA. These extracted features can then serve as input for machine learning classifiers to capture nonlinear relationships among biomarkers, thereby improving patient stratification [118].

As an example, Supervised Deep Generalized Canonical Correlation Analysis (SDGCCA) has outperformed traditional CCA in classifying Alzheimer’s disease and various cancers [116]. When there is a need for biologically interpretable components, nonnegative PCA can enhance the process of discovering molecular patterns [114]. When the aim is to select features across paired omics (e.g., DNA methylation or chromatin accessibility and gene expression), sparse CCA or L2,1-norm-constrained CCA is preferred especially when the number of features exceeds the number of samples [115,117]. For non-linear relationships between two modalities, like enhancer–gene regulation or context-dependent biomarker interactions, kernel CCA and sparse kernel CCA are more suitable options [118]. Thus, standard PCA/CCA is more suitable for initial visualization, while sparse and kernel CCA are more suitable for interpretable biomarker prioritization and nonlinear multi-omics integration [116,118,119].

### 5.2. Matrix Factorization

One of the widely established methods of multi-omics integration are matrix factorization (MF) approaches. These approaches are based on decomposing several data matrices into a collection of common and modality-specific latent factors [120,121]. MF models, including Multi-Omics Factor Analysis (MOFA) and Non-negative Matrix Factorization (NMF), are designed to identify low-dimensional representations capturing the highest variance of the various omics layers. These approaches can also be applied to identify synchronized biological processes, including signaling pathways reflected across both DNA methylation and gene expression [120]. For single-cell data, Joint Semi-orthogonal Non-negative Matrix Factorization (JSNMF), enables the joint analysis of chromatin accessibility (scATAC-seq) and gene expression (scRNA-seq) [121]. JSNMF uses a consensus graph fusion strategy to handle modality-specific noise and align the multiple omics layers more closely to allow accurate cell-type recognition [121]. Matrix factorization methods are generally considered interpretable, as the weights (or loadings) of the latent factors can often be directly associated with specific genes or epigenomic regions. These analyses provide clear biological insights into the underlying patterns captured by the model. They also tolerate missing data in any or all modalities.

However, MF methods normally depend on linear assumptions which cannot necessarily represent complex, nonlinear interactions between different omics layers [122]. MOFA+ is useful for the unsupervised exploration of heterogeneous bulk multi-omics data, as its algorithm distinguishes between shared sources of variation and modality-specific factors. This capability allows researchers to more easily separate meaningful biological structures from technical noise. In contrast, DIABLO, part of the mixOmics framework, is better suited for supervised contexts such as classification and biomarker identification, where sparse cross-modal feature selection is essential. Ultimately, standard matrix factorization methods are valuable when the primary goal is to discover latent structures, whereas sparse supervised variants are preferable when the priority is identifying interpretable multi-omics signatures associated with specific clinical phenotypes [93,94].

### 5.3. Network-Based Fusion

Network-based approaches assume that multi-omics data can be represented as a graph of interlinked nodes (samples or features) and leverage graph-theoretical methods to integrate and analyze the relationships across different omics layers. Similarity Network Fusion (SNF) constructs individual sample-similarity networks for each omics modality and recursively merges them into a single, comprehensive network. The method is especially useful for identifying coherent clusters of samples (e.g., disease subtypes) that are supported by multiple layers of molecular evidence [95].

Integration through Graph Convolutional Networks (MOGONET) is an enhanced network design. It uses Graph Convolutional Networks (GCNs) to learn omics-specific representations, treating samples as nodes within a patient similarity network. The final step of integration is performed using a View Correlation Discovery Network (VCDN), which identifies higher-level correlations in the label space between mRNA expression, DNA methylation and miRNA data [97]. Similarly, DeFusion is another integrative framework based on denoised network regularization to combine multi-omics data. DeFusion enhances the strength of the biomarker discovery and classification tasks by removing technical noise and focusing on the shared topological structure of the networks [123,124].

Another important network-based method, TransNet (Transkingdom Network), is grounded in a systems biology framework, specifically designed to identify causal components underlying host–microbiota interactions by integrating multi-omics data across different biological domains. The method combines various biological data from multiple kingdoms, including both host and microbial organisms, to build extensive interrelationships within the biological system. The accurate analysis of these integrated networks opens the possibility of identifying some influential nodes or components that stimulate the host–microbiota dynamics. The approach is grounded in a comprehensive systems biology and computer network analysis of host–microbiota interactions, enabling the identification of key targets that can be leveraged for therapeutic interventions or mechanistic knowledge [125].

Other network-based approaches are especially helpful in situations where the objective is to deduce regulatory relationships rather than simply clustering samples. As an example, WGCNA (Weighted Gene Co-expression Network Analysis) can be applied to correlate transcriptional modules with epigenetic changes including chromatin accessibility and DNA methylation. This enables researchers to connect global regulatory shifts directly to integrated gene-expression programs. Similarly, the PANDA (Platform for Architecture-Neutral Dynamic Analysis) and NetBID (Network-based Bayesian Inference of Drivers) frameworks are valuable for identifying candidate master regulators or hidden drivers through the combination of regulatory-network structure with multi-omics measurements. Such approaches are particularly applicable when the analysis prioritizes mechanistic explanation and regulatory hierarchies over predictive classification [102,103,104]. In practical terms, WGCNA is better for finding co-expression modules and linking module eigengenes to epigenomic or clinical traits. On the other hand, PANDA and NetBID are better for regulatory-network inference, analyzing transcription-factor activity, and discovering potential master regulators (Table 3).

### 5.4. Deep Learning Frameworks

Deep learning (DL) has completely transformed multi-omics analysis by offering adaptable architectures capable of capturing highly nonlinear interactions among high dimensional data. Variational Autoencoders (VAEs) and multimodal autoencoders are commonly applied in unsupervised integration. These models learn compressed, nonlinear latent space that can simultaneously reconstruct multiple omics layers. This makes them effective for data denoising and the imputation of missing values [96].

With the advent of spatial omics technologies, recent models, such as SpatialFuser, have been developed to combine spatial transcriptomics with other modalities. SpatialFuser is a deep learning-based method designed to preserve spatial context during multi-omics data integration, enabling researchers to investigate how epigenetic regulation varies across tissue architecture [126]. Additionally, Multimodal Factorization AutoEncoder (MAE) combines multi-omics data with already known biological interaction networks. Using a combination of matrix factorization and deep autoencoders, MAE can find statistically significant, yet biologically meaningful features based on the available knowledge bases to enhance interpretability of the results [124,127].

Another sophisticated machine learning approach, ATHENA can find complex interactions among many levels of genomic data which correlate with cancer clinical outcomes, through Grammatical Evolution Neural Networks (GENNs). This method mitigates the problems in predictions based on gene expression. These predictions often focus on single genes and overlook complex interactions or data from different genomic layers like genome, epigenome, transcriptome and proteome. In a study involving ovarian cancer data from The Cancer Genome Atlas (TCGA), ATHENA identified both intra-level and inter-level genomic interactions predictive of patient survival. The application of ATHENA to integrate multiple levels of genomic data achieved a balanced accuracy of 72.89% [128], outperforming models that relied on single-omics data.

Further methodological attention is needed for single-cell multi-omics integration due to unique challenges of the data sparsity, high dimensionality, and the frequent lack of congruence between transcriptomic and chromatin accessibility profiles. When working with paired datasets, such as those generated by 10×Multiome, the Seurat/Signac framework provides a robust solution. Using weighted nearest neighbor (WNN) analysis, it enables joint clustering and the direct association of chromatin accessibility with gene expression at the single-cell level [105].

In contrast, LIGER is especially convenient with unpaired datasets or in systems with strong batch effects across different platforms. Its integrative non-negative matrix factorization (iNMF) algorithm is designed to align and harmonize datasets where measurements were not taken from the same individual cells [106]. Consequently, the selection of these tools depends not only on the omics modalities involved but also on whether the data are paired or unpaired and the degree of technical heterogeneity between experiments.

More generally, multimodal autoencoders and VAE-based models, like OmiVAE, can be effectively applied when the task is to model highly complex nonlinear relationships in large, high-dimensional cohort-level data sets. These methods are preferred when predictive performance—such as survival prediction or subtype classification—is the primary objective. However, they typically offer reduced interpretability compared to factor-based or network-based methods. Consequently, they are appropriate when the dataset size is substantial and predictive accuracy is prioritized over feature-level explainability [96,107]. Practical guidance for selecting deep learning architectures in epigenomic and multi-omics analysis is summarized in Table 5.

### 5.5. Challenges and Practical Considerations in Multi-Omics Data Integration

The integration of epigenomics with other omics data introduces a distinct set of practical challenges that extend beyond those encountered in single-assay analysis. Data harmonization is a key practical issue in all integration strategies. Multi-omics datasets also vary considerably in scale, distribution, sparsity, missing data rates, and platform-specific noise; therefore, they can only be directly compared after rigorous normalization and batch correction. These issues are particularly critical when integrating epigenomic assays, where assay-specific signal architecture and feature definitions of regulatory features often do not align naturally with transcriptomic or proteomic data. Consequently, integration methods must be selected not only for their predictive power but also for their ability to manage heterogeneous feature spaces, technical variability, and incomplete cross-modal data [14,95,129,130].

One of the key challenges lies in the normalization of data across different platforms, given that different technologies produce widely varying data scales and noise characteristics. The purpose of normalization is to ensure that the data in one dataset can be compared to the data in other datasets and minimize technical bias, although inconsistency usually persists [129,131]. Another important challenge is batch effects-systematic bias generated from variability in sample preparation, sequencing procedures, or laboratory conditions can hinder true biological variation. Addressing these batch effects without removing true biological signals requires advanced computational methods [129,130].

## 6. Epigenomics in Disease and Cancer Biology: Clinical and Translational Applications

### 6.1. Epigenomics in Cancer: Mechanisms, Biomarkers, and Therapeutic Opportunities

Recent research has demonstrated how the tumor microenvironment can remodel the epigenome, thereby contributing to cancer progression and metastasis [132]. Epigenetic alterations of DNA methylation, histone modification and RNA nucleotide modification (N6-methyladenosine, m6A) are involved in the progression of lung cancer, pulmonary diseases and can be studied using recent developments in epigenomic technologies [133,134]. Imbalances in small molecules associated with RNA epitranscriptomic machinery can disrupt RNA metabolism including transcription and stability, thereby contributing to uncontrolled cell proliferation and metastasis. Such advances in RNA sequencing technologies allow high-resolution profiling of these RNA modifications, enabling researchers to gain deeper insights into their roles and potentially uncover novel therapeutic molecules in lung diseases [135].

One notable example is the application of an epigenomic analysis to cell-free tumor DNA in ovarian cancer, where epigenomic profiles of DNA methylation combined with artificial intelligence (AI) achieved near-perfect diagnostic value (AUC up to 1.00), exceeding the accuracy of conventional serum markers and imaging [136]. The near-perfect performance should, however, be interpreted with care, as there is a risk that models trained and tested on limited or non-independent cohorts may overestimate the diagnostic accuracy. So, before these biomarkers can be regarded as clinically reliable, independent validation in large, diverse, prospective cohorts is needed. Identified epigenomic signatures offer biologically realistic interpretations of cancer-related pathways, providing greater diagnostics reliability over conventional morphological or protein-based approaches [136]. Additionally, Kim et al. [137] reported that epigenetic markers in urine of patients with bladder cancer exhibit higher sensitivity than conventional cytology for bladder cancer detection. This enhanced sensitivity arises from the ability to detect promoter hypermethylation of tumor suppressor genes, which are associated with tumor development and prognosis [137].

Epigenomics also offers new prospects in disease prevention and personalized medicine. Isothiocyanates and natural substances with epigenetic-modifying properties have been examined in their capacity to prevent the early-stage cancers including skin, colon, lung, breast, and prostate cancers. These agents provide antioxidative, anti-inflammatory, and epigenetic effects, potentially slowing cancer progression and metastasis by modulating epigenomic machinery [138].

Recent research suggests that noncanonical histone modifications also play a role in renal protection. Histone β-hydroxybutyrylation has been demonstrated to act as a mechanism through which β-hydroxybutyrate produces reno-protective effects in the Dahl rat, which demonstrates a mechanistic association between metabolic state and epigenetic regulation in kidney disease [139]. Similarly, epigenetic drugs that target DNA methylation and histone modification are under development to be applied in therapeutic management of cancers like cervical cancer. These drugs are aimed to enable earlier diagnosis, provide rich and functional molecular information on cancer states, reduce dependence on invasive sampling and subjective interpretation, and provide better patient stratification and disease surveillance [1].

Techniques to study single-cell epigenomics have further enhanced the overall understanding of cancer biology, allowing insights into cancer progression, treatment response and mechanisms of relapse [140]. Researchers have effectively used epigenomic signatures to identify the tissue of origin in cancers of unknown primary (CUP), a clinically significant phenotype. These signatures improve diagnostic specificity and guide therapeutic decision-making [141]. Epigenetic changes that disrupt genes and pathways can elucidate tumorigenesis and have been used to develop specific epigenetic therapeutics that help detect minimal residual disease [142,143]. Minimally invasive approaches for cancer screening, early-detection, tissue-of-origin, and disease monitoring technologies have become possible due to the development of cell-free DNA (cfDNA) technologies, mainly based on epigenetic signatures in fluid bio-samples [143]. Another benefit of epigenomic profiling is that it avoids the shortcomings of circulating tumor DNA approaches, which primarily capture genetic mutations but often fail to reflect dynamic transcriptional programs and epigenetic modifications that regulate cancer phenotypes. Immunoprecipitation-based methods capable of detecting histone modifications and DNA methylation in liquid biopsies enable the identification of drug targets, resistance mechanisms and cancer subtype classification using plasma samples alone. This provides important information not accessible through conventional tissue biopsies or genetic testing approaches [144].

As an example, 5hmC profiling of cfDNA has become a novel instrument of oncology with precision because it mirrors cancer biology, such as initiation, progression, and metastasis in diverse types of cancer. Genome-wide 5hmC profiling provides sensitive screening, diagnostics, and prognostic analysis of cancer, whereas traditional methods are only limited to tissue biopsies [145,146]. The identification of epigenetic alterations along with approval of epigenetic drugs like DNA methyltransferase (DNMT) and histone deacetylase (HDAC) inhibitors, represents a paradigm shift in the therapeutics field. These epigenetic drugs target aberrant epigenetic mechanisms and are undergoing extensive clinical evaluation across multiple cancer types [1,147].

### 6.2. Epigenomics Beyond Cancer: Applications in Other Diseases

Beyond cancer, epigenomics plays an important role in other diseases, including stroke and cardiometabolic disorders, by influencing gene regulation and contributing to disease onset, progression, and patient outcomes [148]. Environmental and lifestyle changes can also cause epigenetic alterations in cardiometabolic disease, which influence the presentation of the disease. The deciphering of these epigenetic signatures may reveal novel molecular targets for precision medicine, enabling strategies to minimize cardiovascular risk [149].

One of the most significant advances has been the development of epigenetic clocks based on DNA methylation patterns. These tools have enhanced the molecular-level investigation of aging processes and age-related diseases, providing more precise biomarkers of biological age. Moreover, these tools provide insight into how environmental exposures such as nutrient imbalances, particularly in B-vitamins, and polymorphisms in one-carbon metabolism pathways impact epigenetic aging and disease susceptibility. These findings highlight the intersection of nutritional epigenomics and health outcomes [150].

A recent disease-focused example is the study by Kiliçarslan et al. in Biomedicines, which validated DNA methylation of two genes, namely LDHC and SLC35G2, as potential epigenetic markers for food allergy using machine learning and deep learning techniques. Their study adopted differential methylation analysis along with several classifiers, such as support vector machine, k-nearest neighbors, random forest, artificial neural network, and stacked autoencoder models. Importantly, the biomarkers were also tested on an external validation dataset, and the results were reproducible [151]. These results demonstrate the potential of computational validation of disease-specific methylation signatures for precision diagnostics in allergy.

Advances in chromatin biology in neuroscience have led to changes in epigenetic processes. These processes can regulate the expression of genes in neurons, and this is important for synaptic plasticity and memory. In addition to histone phosphorylation and acetylation, recent findings have highlighted that DNA and histone methylation also play crucial roles in behavior and long-term memory regulation [152]. The study of the interactions between epigenetic remodeling and transcription factor activity can provide important insights into the mechanisms by which transcriptional regulation governs memory storage [152]. In the cardiovascular biology of heart failure, recent research indicates that chromatin-modifying enzymes and the 3D structure of the genome, including the lamina-associated domains, play important roles in the epigenetic regulation of gene expression, influencing disease pathology. Recent progress has revealed that histone demethylases and deacetylases play key roles in regulating fetal gene expression programs and cardio protection and that epigenetic responses to stress and external stimuli can determine whether the outcome is adaptive or pathological [153]. In cardiovascular disease, Zhang et al. developed a model to predict coronary heart disease using methylation-regulated genes identified in the Framingham Heart Study, showing the potential of machine learning-based DNA methylation biomarkers in cardiovascular disease [154]. Other machine learning applications have been used in cancer, such as the discovery of biomarkers for stomach cancer using integrative DNA methylation and gene expression analyses and the identification of pan-cancer methylation markers using deep learning [155,156]. However, machine learning-based methylation biomarkers need to be better externally validated, reported systematically, and controlled for overfitting before being translated to clinical use [157].

Beyond cancer, translational research in epigenomics has also been applied to mental health and neurodevelopmental disorders, where epigenetic processes play critical roles in brain development and function [158].

The combination of SNPs and transcriptomics along with DNA methylation has been especially beneficial for metabolic studies. In a recent study, the DIABLO (Data Integration Analysis for Biomarker discovery using Latent cOmponents) algorithm (of the mixOmics R package) was employed to accurately predict Type 2 Diabetes (T2D) using several omics layers from human pancreatic islets. This integrative examination identified novel candidate biomarkers, including the SACS (Sacsin Molecular Chaperone), TXNIP (Thioredoxin-interacting protein) DNA methylation and OPRD1 (Opioid Receptor Delta 1) expression, that provide insight into biological processes of mitochondrial dysfunction and altered insulin secretion in T2D [159].

Epigenetic clocks—models that estimate biological age or health condition based on DNA methylation patterns—have reached new levels of resolution and accuracy. BS-clock is a recently developed DNA methylation clock optimized using high-resolution bisulfite sequencing data, enabling more precise predictions of human aging compared to the earlier array-based models [160]. Moreover, iTARGET (Interpretable Tailored Age Regression of Grouped Epigenetic Traits) employs explainable boosting machine models to generate interpretable age predictions, revealing age-specific CpG interactions that were previously hidden [161]. These predictors are increasingly applicable in personalized medicine and can be used as biomarkers of disease risk and environmental exposure. However, current clocks work best in blood, and there are significant problems in other tissues, or in non-European populations [162]. Moreover, recent research shows that bioinformatic tools can help identify therapeutic targets for certain chronic diseases. As an example, DNA methylation of SACS in Type 2 Diabetes has been suggested as a potential target for restoring mitochondrial function in the future [159]. Regarding respiratory diseases, DNA methylation changes associated with asthma have been identified using tools like easyEWAS, which facilitate the epigenetic analysis of how environmental exposures impact lung health [57].

### 6.3. Clinical Translation: Current Progress, Standardization, and Implementation Challenges

Epigenomics has important clinical and translational applications in advancing the process of diagnosis, prognosis, and personalized therapy in various medical fields. One of the most significant challenges in the rapidly evolving epigenetics field is standardization, which has been addressed through several updated or newly developed R and Bioconductor packages. For example, easyEWAS is a user-friendly software tool that combines the latest approaches to Epigenome-Wide Association Studies and accounts for the position-specific or region-specific analysis of differential methylation [78]. The BISulfite-seq Command line User Interface Toolkit (with the biscuiteer R package) is a standards-compliant tool suite that simultaneously performs genetic variant calling and DNA methylation inference in both bulk and single-cell analyses [163]. Moreover, TENET is an epigenetic-based network tracing algorithm that determines key regulatory entities and transcription factors across ten cancer types in pan-cancer studies [164]. As summarized in Table 6, several epigenomic discoveries have already entered clinical practice through FDA-approved diagnostic assays and therapeutic agents. However, broader adoption still requires standardization, prospective validation, cost-effectiveness, and compatibility with existing clinical workflows.

Despite these promising uses in early cancer detection, personalized medicine, and biological age estimation, the field of epigenomics faces significant hurdles that must be addressed to ensure successful clinical implementation. Reports of high diagnostic or prognostic performance derived from small patient cohorts should be interpreted with caution unless validated in large, diverse, and independent datasets. In practice, clinical adoption depends on multiple factors, including robust model performance, assay standardization, and reproducibility across laboratories. Additional considerations include sample quality, affordability, turnaround time, scalability for routine use, effective control of batch effects, and overall cost-effectiveness. Moreover, regulatory approval, prospective validation in real-world clinical settings, and the limited interpretability of certain machine learning models remain significant barriers to implementation in clinical decision-making. Therefore, the path from computational epigenomic discovery to clinical application requires not only the identification of reliable biomarkers but also rigorous technical validation, multicenter studies, and clear demonstration of clinical utility beyond retrospective performance metrics [165,166,167].

## 7. Discussion—Current Limitations and Future Directions

**Figure 6:**
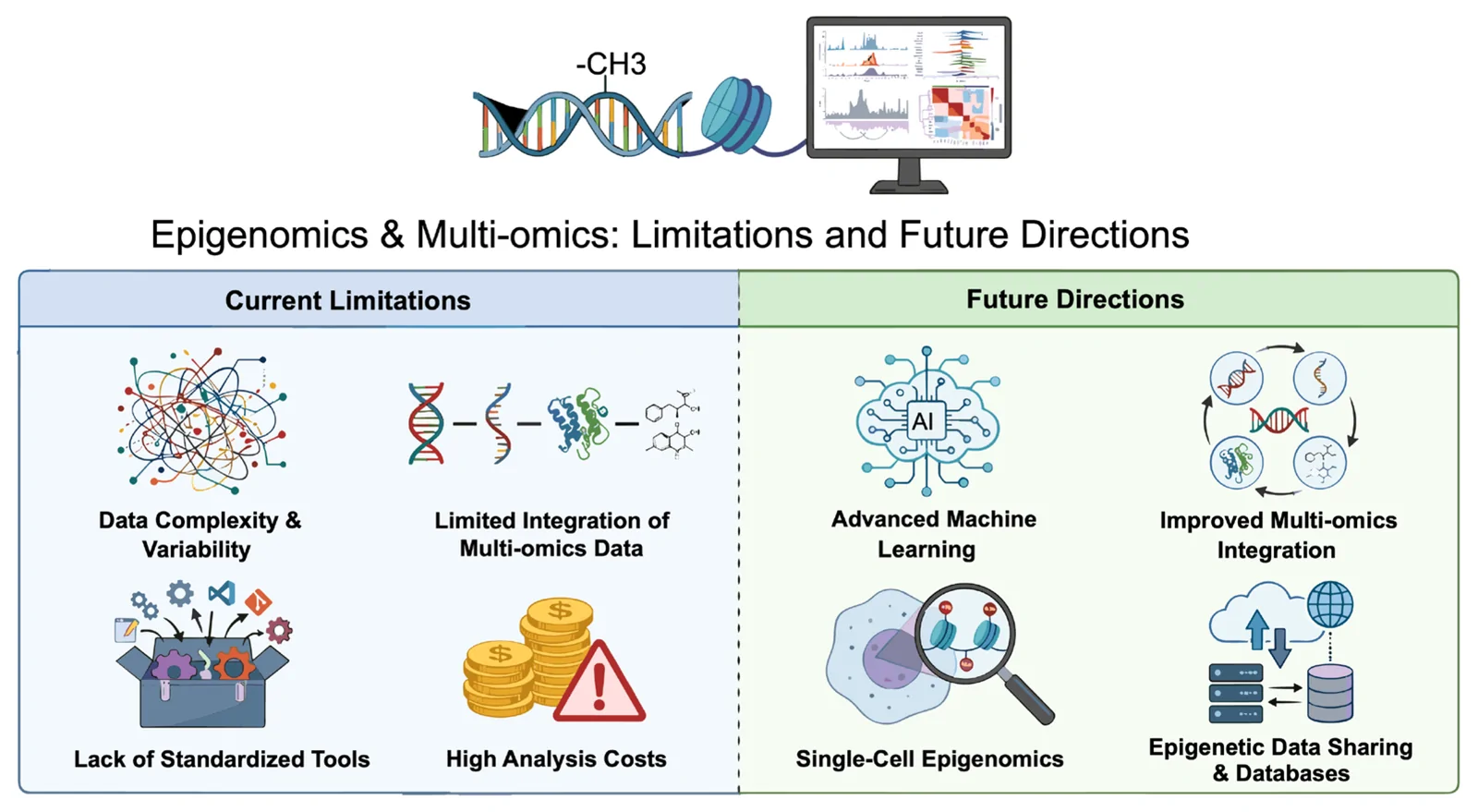
Current challenges and future horizons in epigenomics. A comparative overview highlighting the existing bottlenecks in epigenomic research versus the emerging strategies designed to overcome them. The figure summarizes the field’s evolution from high-cost, fragmented analysis toward a more integrated and accessible future.

Although significant progress has been made in epigenomic technologies and computational tools, there are several challenges that continue to hinder the reproducibility, interpretation, and clinical translation of epigenomics. Some of these include assay-specific tools, normalization differences, batch effects, sparse single-cell data, cross-platform heterogeneity, non-coding region annotation, and limited model interpretability. These broader challenges are conceptually summarized in Figure 6. Due to the detailed discussions of these issues in the previous sections, this section focuses on how these challenges can be addressed with future research including standardized workflows, transparent parameter reporting, benchmarked computational tools, independent validation data, and biologically interpretable AI models.

Epigenomic regulation is also biologically complex, posing major interpretive challenges. Numerous epigenetic changes occur outside well-annotated protein-coding regions, making the functional annotation of regulatory elements difficult. The combination of epigenomic datasets with 3D genome structure, enrichment analysis, and machine learning approaches is actively pursued to infer functional significance; however, a definitive interpretation remains complex [168]. Causality or association in epigenetic studies is also difficult to measure, particularly considering interaction between genetic and epigenetic variables influencing variation at the population level. It can be seen in the research of diseases and traits, where it is very difficult to separate the influence of epigenetics and genetic variation that forms the background [169]. The example of mitochondrial retrograde signaling illustrates how organelle-to-nucleus communication can lead to epigenomic remodeling. Metabolites like acetyl-CoA, α-ketoglutarate, succinate, and NAD+/NADH are influenced by changes in mitochondrial activity and can affect histone acetylation, DNA/histone demethylation, and sirtuin-dependent chromatin regulation. Hence, some epigenomic changes may be due to metabolic stress or mitochondrial dysfunction, and not necessarily nuclear events. Adopting this approach could facilitate the transition of future integrative studies from association-based observations to testable causal models [170]. Epigenomic research is also complicated by technological and ethical issues. The quality of samples used in an experiment and assay biases are experimental factors that influence the reliability of data. Furthermore, ethical concerns regarding privacy, possible discrimination due to epigenetic data, and fair access to clinical uses of epigenetics remain important questions regarding the translation of epigenomics into personalized medicine [171].

Artificial intelligence (AI) has revolutionized epigenomic data analysis by providing computational models capable of handling the scale, complexity, and regulatory interdependence inherent in epigenetic systems. The critical literature suggests that traditional analyses—often reliant on linear models and manual feature engineering—fail to capture the intricate relationships between DNA methylation, histone modifications, chromatin accessibility, and 3D genome organization [172,173]. Conversely, modern machine learning (ML) and deep learning (DL) architectures, such as CNNs, GNNs, and transformer-based models, can detect patterns directly from raw sequencing reads and processed epigenomic profiles. These frameworks facilitate the precise estimation of chromatin states, promoter-enhancer interactions, and transcriptional activity [173,174]. Furthermore, the integration of unsupervised and self-supervised learning enables the discovery of novel regulatory programs without the need for extensive labeled datasets. This approach maximizes the utility of large-scale consortia data [172].

Despite these advancements, several major challenges continue to hinder the clinical integration of AI-based epigenomic tools. Model interpretability remains a primary obstacle; many deep learning frameworks function as ‘black boxes’ that fail to provide the biological insights necessary to instill confidence in clinicians in diagnostic or prognostic results [172,175]. Furthermore, a lack of generalizability and inherent biases in training data often arise because models are trained on datasets unrepresentative of diverse populations, cancer subtypes, or disease stages. This frequently leads to reduced performance when tools are applied to independent cohorts or real-world clinical contexts [172].

Recent research has tried to address these limitations using explainable AI (XAI) tools, such as SHAP (SHapley Additive exPlanations), which provides a quantitative importance score to each of the input features for a particular model prediction. LIME (Local Interpretable Model-Agnostic Explanations) employs locally faithful linear approximations around individual predictions, enabling interpretable explanations of classifier decisions. Biologically grounded architectures further enhance transparency and ensure that model predictions are biologically relevant [175,176]. Standardized benchmarking protocols and the use of large-scale consortia, such as ENCODE, TCGA, and Roadmap Epigenomics, are essential for improving reproducibility and enabling meaningful comparisons across studies [172]. Finally, rigorous clinical validation, multi-center trials, and external cohort assessments are required to demonstrate the robustness and utility necessary for regulatory approval [165].

This study is a narrative review and thus has some methodological limitations. Selection bias was possible due to the literature selection process, as the literature was selected based on relevance rather than a formal PRISMA-style screening process. Moreover, the search was conducted primarily in PubMed and Google Scholar databases and only on articles published in the English language, which may have missed relevant articles from other databases and languages. Hence, this review is not a systematic review, but rather a critical narrative review.

## 8. Conclusions

Advances in epigenomics are primarily driven by the evolution of experimental technologies and increasingly sophisticated computational methods for data processing and interpretation. A central conclusion of this review is that no single methodology is universally optimal. Instead, the selection and integration of tools must be tailored to the specific assay type, data structure, and biological objective, while maintaining an appropriate balance between interpretability, scalability, and predictive performance. Nevertheless, persistent challenges—including issues with reproducibility, normalization, non-coding region annotation, platform harmonization, and clinical validation—remain significant barriers to robust biological discovery.

Emerging frontiers, such as single-cell epigenomics, cross-platform data fusion, and explainable AI (XAI), are shifting the field from descriptive profiling toward mechanistic and clinically actionable models of gene regulation. This review underscores that future progress requires more than the mere accumulation of data; it necessitates the development of transparent, benchmarked, and biologically informed computational frameworks. Only through such rigor can these frameworks facilitate reproducible discoveries that are reliable enough for routine clinical implementation.

## Figures and Tables

**Table 1:** Epigenomics tools organized by analysis workflow. Examples of bioinformatics tools for genomic tasks, including applications, input/output formats, advantages, and limitations. The table tracks the progression from raw sequencing data to functional biological insights across various high-throughput assays.

Workflow Step	Tool and Source	Assay/Data Type	Methodology/Analytical Principle	Input/Output	Strengths/Limitations	Decision Guidance
Quality control/read-quality assessment	FastQC; (v0.12.0) (Babraham Bioinformatics)	All sequencing-based assays	Evaluates per-base quality, GC content, duplication, adapter contamination, and overrepresented sequences.	FASTQ → QC report	Fast and widely accepted. does not correct or filter reads.	Run on raw reads before trimming or alignment; use Trim Galore or fastp to address issues.
QC report aggregation	MultiQC; (v1.35) (open-source project)	All assays	Aggregates QC metrics from multiple tools into one sample-level report	QC outputs → combined report	Excellent for multi-sample studies. depends on upstream QC tools	Use when comparing QC metrics across many samples, replicates, or assay types.
Alignment and methylation calling	Bismark; (v3.1.0) (Babraham Bioinformatics)	WGBS, RRBS, bisulfite-seq	Bisulfite-aware alignment and cytosine methylation calling across CpG, CHG, and CHH contexts	FASTQ → BAM + methylation calls	Accurate and well established; computationally intensive	Preferred for high-confidence bisulfite alignment and standard methylation-calling workflows
Alignment and methylation mapping	BS-Seeker2; (v2.1.0) (open-source)	Bisulfite-seq	Bisulfite-read alignment using supported short-read aligners followed by methylation extraction	FASTQ → BAM + methylation calls	Faster than Bismark in some settings; less commonly adopted	Use when runtime is the main constraint or when aligner flexibility is needed
Peak calling/signal detection	MACS2; (v 2.2.9.1) (open-source peak caller)	ChIP-seq, ATAC-seq	Model-based peak calling using local background enrichment and statistical significance testing	BAM → peak BED files	Dynamic local background modeling improves sensitivity in regions with uneven coverage; it may be less suitable for broad marks.	Best for TF ChIP-seq and ATAC-seq with sharp profiles; use caution with --nomodel because incorrect shift/fragment-size assumptions can affect peak position and sensitivity; validate results.
Broad peak detection	SICER; (open-source command-line tool)	ChIP-seq	Identifies spatially clustered enriched regions suitable for broad histone marks	BAM → broad peak regions	Good for broad marks; less sensitive for narrow peaks	Use for broad histone modifications such as H3K27me3 or H3K9me3
Differential methylation analysis	methylKit; (v1.26.x) (Bioconductor R package)	Bisulfite-seq	Statistical comparison of methylation levels across samples or conditions	Methylation calls → DMRs + plots	Flexible experimental designs; sensitive to low coverage	Use after reliable methylation calling to compare methylation changes between groups
Differential chromatin binding analysis	DiffBind; (v3.20.0) (Bioconductor R package)	ChIP-seq	Uses count-based statistical frameworks such as DESeq2 or edgeR to identify differential binding	Peak files + BAM → differential peaks	Supports complex designs; memory intensive for large datasets	Use when testing whether chromatin binding or histone-mark enrichment differs between conditions
Motif and regulatory analysis	HOMER; (v5.1) (HOMER software suite)	ChIP-seq, ATAC-seq	Motif enrichment and regulatory sequence analysis within peak regions	Peak files → enriched motifs	Provides strong motif discovery; background selection affects results.	Use after peak calling to infer candidate transcription factors or regulatory motifs
ATAC-seq-specific quality control	ATACseqQC; (v1.37.1) (Bioconductor R package)	ATAC-seq	Evaluates ATAC-specific QC metrics such as fragment-size distribution, nucleosome pattern, TSS enrichment, and mitochondrial reads	BAM → QC metrics + diagnostic plots	Tailored to ATAC-seq; not a peak caller	Use before downstream peak calling or accessibility analysis to confirm library quality
Single-cell chromatin accessibility analysis	ArchR; R package/(open-source (v1.0.3) GitHub project)	scATAC-seq	Scalable framework for clustering, gene-score estimation, peak calling, trajectory analysis, and integration	Fragment files → clusters, peaks, gene scores	Powerful and scalable; computationally demanding	Use for large scATAC-seq projects requiring broad end-to-end analysis
Large-scale single-cell accessibility analysis	SnapATAC2; (v2.10.0) (Python package in the scverse ecosystem)	scATAC-seq	Constructs cell-by-bin matrices and performs dimensionality reduction, clustering, and trajectory analysis	FASTQ/BAM/fragment files → clusters, embeddings, trajectories	Handles large datasets; workflow can be complex	Use when bin-based feature representation is preferred or when memory efficiency is critical
Variant-to-cell-state interpretation	SCAVENGE; (v2.0.1) (open-source software associated with GitHub)	scATAC-seq + GWAS/fine-mapped variants	Uses variant enrichment, cell–cell similarity networks, and network propagation to assign trait relevance scores to cells	scATAC-seq matrix + variant data → prioritized cells/cell states	Links genetic risk to regulatory cell states; requires variant input and is not a preprocessing tool	Use when the goal is to identify disease- or trait-relevant cell types/states from scATAC-seq and GWAS data
Sparsity correction/signal enhancement	scCASE; (v3.1.0) Python (package/GitHub)	scATAC-seq	Enhances sparse chromatin accessibility matrices using matrix-factorization-based signal recovery	Sparse scATAC-seq matrix → enhanced accessibility matrix	Improves sparse signal; possible imputation bias if not validated	Use when dropout or low coverage limits clustering, visualization, cell-state identification, or integration
Single-cell chromatin accessibility embedding/feature representation	CASTLE; (v3.2.0) (PyTorch-based Python package/GitHub)	scCAS/scATAC-seq	Learns discrete latent representations of sparse single-cell chromatin accessibility profiles to enable cell-state characterization and dimensionality reduction	Sparse scATAC-seq matrix → discrete latent embeddings + cell-state patterns	Provides interpretable latent representations; requires model training and is not a general preprocessing or peak-calling tool	Use for interpretable discrete cell-state embedding; pair with other tools for peak calling or RNA integration
Genomic interval operations	BEDTools; (v2.28.0) (open-source command-line suite)	Region-based genomic assays	Performs interval intersection, overlap, merging, subtraction, and coordinate-based comparison	BED/BAM/GFF → overlaps or filtered regions	Fast and flexible; command-line expertise needed	Use for essential genomic-region operations such as intersecting peaks with genes, promoters, or enhancers
Peak annotation	ChIPseeker; (v1.40.0) (Bioconductor R package)	ChIP-seq, ATAC-seq, peak-based assays	Annotates peaks relative to genes and genomic features and visualizes peak distributions	BED peak files → gene annotations + plots	Easy R/Bioconductor integration; nearest-gene annotation can be misleading for distal elements	Use for rapid gene-centered annotation and peak-distribution analysis
Regulatory-region enrichment	GREAT; (v2.12.2) (web-based platform from the McLean Lab at Stanford)	ChIP-seq, ATAC-seq, regulatory regions	Links noncoding regions to putative target genes using proximal and distal regulatory domains	Peak files → functional enrichment results	Better for distal regulatory interpretation, depends on predefined association rules	Use when interpreting noncoding regions where nearest-gene annotation is insufficient

**Table 2:** Comparative analysis of widely used DNA methylation sequencing technologies. Comparison of common laboratory methods for profiling DNA methylation, categorized by their underlying biochemical principles, practical benefits, and technical constraints.

Method	Principle	Advantages	Limitations
WGBS	Bisulfite conversion and sequencing of almost the entire genome	Considered the gold standard for unbiased methylome profiling. Single-base resolution and near-comprehensive genome-wide coverage can be achieved.	Expensive, and bisulfite treatment may degrade DNA [50].
RRBS	Restriction enzyme enrichment of CpG-rich regions followed by bisulfite sequencing.	More cost-effective than WGBS. High resolution in CpG islands and promoters. Lower sequencing requirement.	Covers only a subset of the genome; biased toward CpG-dense regions; may miss intergenic/distal regulatory CpGs [50].
MSP	PCR amplification of bisulfite-converted DNA using primers specific for methylated sequences.	Fast, inexpensive, highly sensitive, and suitable for low-input samples.	Limited to predefined loci and not genome-wide. Also, it depends heavily on primer design. It may provide only qualitative or semi-quantitative information [51,52].
Illumina EPIC/EPIC v2 arrays	Array-based interrogation of predefined CpG sites	Cost-effective for large cohorts; widely used in biomarker and epigenetic-clock studies	Limited to selected CpG probes; cannot detect all methylation sites [39,40]
Targeted bisulfite sequencing	Bisulfite sequencing of selected genes or regions	High-depth validation of candidate biomarkers; useful for clinical assay development	Restricted to selected regions; requires careful target and primer design [44]
cfDNA methylation profiling	Methylation analysis of circulating cell-free DNA from blood or plasma.	Useful for liquid biopsy, early cancer detection, tissue-of-origin prediction, and disease monitoring	Low tumor fraction; pre-analytical variation; requires strong validation [41,42,43]
Nanopore/PacBio methylation detection	Direct detection of DNA modifications from native long-read sequencing	Detects methylation without bisulfite conversion; useful for repetitive regions, haplotypes, and structural variants	Accuracy depends on sequencing depth, chemistry, and modification-calling algorithms [45,46]
Single-cell methylome sequencing	Single-cell or single-nucleus bisulfite/methylcytosine sequencing	Reveals cell-to-cell methylation heterogeneity; useful in cancer, development, and complex tissues	Sparse data, low coverage, high cost, and complex analysis [47,48,49]

**Table 5:** Practical guidance for selecting deep learning architectures in epigenomic and multi-omics analysis.

Architecture	Best Suited Data Type	Typical Dataset-Size Consideration	Main Use Case	Key Limitation
Standard autoencoder	Dense or moderately sparse bulk multi-omics datasets. DNA methylation, transcriptomics, proteomics, or matched feature matrices.	Can be trained on moderate-sized bulk multi-omics datasets. Stable feature compression improves as sample size increases. Larger matched cohorts are preferable for subtype discovery or prediction to reduce overfitting	(1) Reconstruction-based dimensionality reduction. (2) Denoising (3) Nonlinear feature compression Clustering and subtype prediction.	(1) Less interpretable than factor-based methods. (2) Does not provide uncertainty estimates
Variational autoencoder (VAE)	Sparse, noisy, or missing single-cell and bulk multi-omics data. scRNA-seq, scATAC-seq, and TCGA-style multi-omics datasets	Generally, benefits from large datasets. Single-cell VAE models are stable with thousands of cells. Cohort-level VAE models require sufficient matched samples to avoid unstable latent representations. Very small datasets may increase risk of poor latent-space learning or posterior collapse	(1) Probabilistic latent-space learning. (2) Imputation. (3) Cross-modal representation learning. (4) Uncertainty-aware representation. (5) Prediction	(1) Latent variables may be difficult to interpret. (2) Requires careful tuning and validation.
Graph neural network (GNN)	Network-structured data. Spatial omics, cell–cell graphs, gene–gene graphs, patient-similarity graphs, or regulatory networks	Requires enough nodes and a reliable graph structure. Performance improves when graphs contain sufficient samples, cells, or features for stable neighborhood aggregation. MOGONET was evaluated on TCGA-style cohorts containing hundreds to more than 1000 samples	(1) Modeling relationships among genes, cells, regulatory regions, spatial neighborhoods, or patient-similarity networks. (2) Supervised classification when relational structure is important	(1) Performance depends strongly on graph construction. (2) Prior-network quality can strongly affect results
Multimodal/hybrid autoencoder	Matched multi-omics datasets. DNA methylation + expression + miRNA. Paired scRNA-seq + scATAC-seq	Larger matched multimodal datasets are preferred. The model must learn both within-modality and cross-modality structure. Smaller datasets increase the risk of overfitting and poor generalization	(1) Joint integration (2) Nonlinear feature learning (3) Multimodal reconstruction (4) Classification	(1) Computationally demanding. (2) Often less biologically transparent.

**Table 6:** Representative clinically implemented epigenomic diagnostics and therapeutics.

Clinical Implementation/Translational Significance	Examples	Epigenomic Basis
Methylation-based epigenomic assays have reached clinical use in colorectal cancer screening, including FDA-approved tests such as Epi proColon, Cologuard, Cologuard Plus, and Shield. Epi proColon detects methylated SEPT9 in plasma, Cologuard includes methylated BMP3 and NDRG4 in stool, and Shield is a blood-based cfDNA screening test that uses methylation-related signals	Epi proColon^®^, Cologuard^®^/Cologuard, Plus™, Shield™	Epi proColon detects methylated SEPT9 DNA in plasma. Cologuard detects stool DNA methylation markers including BMP3 and NDRG4; Cologuard Plus detects hypermethylated LASS4, LRRC4, and PPP2R5C. Shield is an NGS-based blood test detecting genomic and epigenomic cfDNA alterations, including methylation and fragmentation patterns.
DNMT inhibitor therapy established DNA methylation as a clinically actionable therapeutic target, especially in myelodysplastic syndromes and related hematologic malignancies.	Azacitidine, Decitabine	Azacitidine and decitabine are nucleoside analogue agents used for MDS and related hematologic malignancies.
HDAC inhibitor therapy demonstrates that histone acetylation pathways are druggable in selected hematologic malignancies and, givinostat, in indications beyond oncology.	Vorinostat, Romidepsin, Givinostat	HDAC inhibitors target histone deacetylase activity and alter chromatin acetylation states. Vorinostat and romidepsin are used for CTCL, whereas givinostat is approved for Duchenne muscular dystrophy.
Targeted epigenetic-regulatory therapy represents precision therapy targeting upstream drivers of epigenomic dysregulation and companion-diagnostic-guided treatment selection.	Enasidenib, Ivosidenib	IDH2/IDH1 inhibitors reduce mutant-IDH–driven 2-hydroxyglutarate production, an upstream cause of abnormal DNA and histone methylation states.

## Data Availability

No new data were created or analyzed in this study. Data sharing is not applicable to this article.

## Abbreviations

The following abbreviations are used in this manuscript:

ATAC-seq	Assay for Transposase-Accessible Chromatin using sequencing
ATHENA	Analysis Tool for Heritable and Environmental Network Associations
CUP	Cancers of Unknown Primary
CASTLE	single-cell Chromatin Accessibility Sequencing data analysis via discreTe Latent Embedding
CfDNA	Cell-free DNA
CoBRA	Combined Bisulfite Restriction Analysis
ChIP-seq	Chromatin Immunoprecipitation sequencing
CCA	Canonical Correlation Analysis
DROMPA	Discrepancy Restricted Overlapping Mapped Peak Analyzer
DL	Deep Learning
DNMT	DNA methyltransferase
DIABLO	Data Integration Analysis for Biomarker discovery using Latent cOmponents
FAIRE-Seq	Formaldehyde-Assisted Isolation of Regulatory Elements
GENN	Grammatical Evolution Neural Networks
GREAT	Genomic Regions Enrichment of Annotations Tool
GCNs	Graph Convolutional Networks
HDAC	Histone Deacetylase
HOMER	Hypergeometric Optimization of Motif EnRichment
HMMRATAC	Hidden Markov ModeleR for ATAC
iTARGET	Interpretable Tailored Age Regression of Grouped Epigenetic Traits
JSNMF	Joint Semi-orthogonal Non-negative Matrix Factorization
LIANTI	Linear Amplification via Transposon Insertion
LIME	Local Interpretable Model-Agnostic Explanations
MeDIP-seq	Methylated DNA Immunoprecipitation Sequencing
MSP	Methylation-Specific Polymerase Chain Reaction
MAPS	Model-based Analysis of PLAC seq
MALBAC	Multiple Annealing and Looping-Based Amplification Cycles
MF	Matrix Factorization
MOFA	Multi-Omics Factor Analysis
MAE	Multimodal Factorization AutoEncoder
MOGONET	Multi-omics Integration through Graph Convolutional Networks
NMF	Non-negative Matrix Factorization
OPRD1	Opioid Receptor Delta 1
PCA	Principal Component Analysis
RRBS	Reduced Representation Bisulfite Sequencing
scWGBS	Single-Cell Whole Genome Bisulfite Sequencing
scRRBS	Single-Cell Reduced Representation Bisulfite Sequencing
scBS-seq	Single-Cell Bisulfite Sequencing
snmC-seq	Single-Nucleus Methylcytosine Sequencing
scATAC-seq	Single-Cell ATAC-seq
ScDEC-Hi-C	Single-Cell deep embedded clustering
SCAVENGE	Single-Cell Analysis of Variant Enrichment through Network propagation of GEnomic data
scCASE	Single-Cell Chromatin Accessibility Sequencing Enhancement
SDGCCA	Supervised Deep Generalized Canonical Correlation Analysis
SNF	Similarity Network Fusion
SACS	Sacsin Molecular Chaperone
SEACR	Sparse Enrichment Analysis for CUT&RUN
SHAP	SHapley Additive exPlanations
TCGA	The Cancer Genome Atlas
TransNet	Transkingdom Network
TXNIP	Thioredoxin-interacting protein
VCDN	View Correlation Discovery Network
VAEs	Variational Autoencoders
WGBS	Whole Genome Bisulfite Sequencing
NetBID	Network-based Bayesian Inference of Drivers
PANDA	Platform for Architecture-Neutral Dynamic Analysis
WGCNA	Weighted Gene Co-expression Network Analysis
iNMF	Integrative Non-negative Matrix Factorization
LIGER	Linked Inference of Genomic Experimental Relationships
